# Supplementary material for: Zero-preserving imputation of single-cell RNA-seq data
Source: Nat Commun. 2022 Jan 11;13:192. doi: 10.1038/s41467-021-27729-z (PMC8752663; doi:10.1038/s41467-021-27729-z)
Supplement: Supplementary file 1 — Supplementary Information [file 41467_2021_27729_MOESM1_ESM.pdf]

Supplementary Information for "Zero-preserving imputation of single-cell RNA-seq data"

The supplement has two parts: 1) Theoretical analysis of ALRA and 2) Supplementary Tables and Figures.

Supplementary Note: ALRA Matrix Perturbation Analysis

In this appendix, we provide theoretical justification for the ALRA procedure using tools from matrix perturbation analysis. We show that after low-rank approximation of the expression matrix, the entries corresponding to true, biological zeros are symmetrically distributed around zero. This result then justifies ALRA's thresholding step, where we use the most negative values in each gene to restore biological zeros after low-rank approximation. For simplicity, the main theorem and proof are presented under a dropout model where entries of the expression matrix are set to zero with a fixed probability. We also show that our results hold under a more realistic multinomial model, where zeros are the result of insufficient sampling of reads in each cell.

1. Problem setup

Let X be an $m \times n$ matrix of rank r, whose entries are all non-negative. In our biological context, the rows represent genes, whereas the columns represent n samples or cells. Thus, X_{ij} is the measured expression of gene i at cell j. As biological cells often simultaneously express only a subset of all genes, we thus assume that each column of X has a non-negligible fraction of zero entries.

Here we consider a case where we do not observe X, but rather, a matrix

$$\tilde{X} = X + E, \tag{1}$$

where the matrix E is random and corresponds to various sources of variability, corruptions and errors introduced by the measurement process. We first analyze in detail a simple model, whereby E represents dropout with a uniform rate p, and then later extend the analysis to a more realistic multinomial model. Under the dropout model, entries of the matrix E are all independent random variables, distributed as follows,

$$E_{ij} = \begin{cases} -X_{ij} & \text{with probability} \quad p, \\ 0 & \text{with probability} \quad 1-p. \end{cases} \tag{2}$$

The low-rank matrix completion problem we consider is to estimate the matrix X given the observed matrix $\tilde{X}$. In the main text we proposed ALRA, an SVD-based approach for this task. In details, denote the SVD of the observed matrix $\tilde{X}$ by

$$\tilde{X} = \sum_{i=1}^{\min(m,n)} \tilde{\sigma}_i \tilde{u}_i \tilde{v}_i^T.$$

where $\tilde{u}_i, \tilde{v}_i$ are the left and right singular vectors and $\tilde{\sigma}_i$ are their corresponding singular values.

Then, ALRA first estimates the rank r, and then computes the rank-r approximation to the observed matrix,

$$\tilde{Z} = \sum_{i=1}^{r} \tilde{u}_i \tilde{v}_i^T \tilde{\sigma}_i. \tag{3}$$

In general, the entries of $\tilde{Z}$ are all not zero. Hence the crucial question, "which cells express a given gene?" cannot be directly answered by this matrix. Moreover, some entries of $\tilde{Z}$ are negative, which is inconsistent with the assumption that all entries $X_{ij} \geq 0$. To provide an estimate $\hat{X}$

of X, the last step of ALRA is to threshold each row (gene) of $\tilde{Z}$ by the absolute value of the $p = 0.001$ quantile in that row.

The purpose of this appendix is to provide a rigorous mathematical and statistical justification for the ALRA procedure. This is described formally in Theorem 1 below, which states that under suitable conditions, the entries which originally were zero in the matrix X are symmetrically distributed around zero in the reconstructed matrix $\tilde{Z}$. We prove Theorem 1 under the model (2), in which the observed non-zero entries contain no noise at all. After Theorem 1, we discuss more realistic models under which Theorem 1 still holds.

2. Theoretical Analysis of ALRA

The first step in our analysis is to rewrite (1) as follows,

$$\tilde{X} = X + E = X + \mathbb{E}[E] + (E - \mathbb{E}[E]) = (1-p)X + \tilde{E},$$

where $\tilde{E} = E - \mathbb{E}[E]$ is a mean-zero random matrix with independent entries, distributed as

$$\tilde{E}_{ij} = \begin{cases} -(1-p)X_{ij}, & \text{with probability } p \\ pX_{ij}, & \text{with probability } 1-p. \end{cases} \tag{4}$$

For future use, we note that the variance of each entry is given by

$$\mathrm{Var}[\tilde{E}_{ij}] = p(1-p)^2 X_{ij}^2 + (1-p)p^2 X_{ij}^2 = p(1-p)X_{ij}^2. \tag{5}$$

Note that entry-wise, $\tilde{E}_{ij}$ is not necessarily small with respect to X_{ij}. However, as we show later, for small p, $\|\tilde{E}\| \ll \|(1-p)X\|$, where $\|\cdot\|$ denotes the spectral norm of a matrix. In other words, from a spectral point of view, the matrix $\tilde{E}$ can be viewed as a small perturbation of the low rank matrix $(1-p)X$. Similar to [8,4], to analyze the properties of the top singular values and singular vectors of $\tilde{X}$, used to construct the matrix $\tilde{Z}$ above, we introduce a continuous parameter $\epsilon > 0$, and study, as a function of ϵ, the singular values and singular vectors of

$$\tilde{X}(\epsilon) = (1-p)X + \epsilon\tilde{E}.$$

We then plug these expressions into the corresponding matrix $\tilde{Z}(\epsilon)$,

$$\tilde{Z}(\epsilon) = \sum_{i=1}^{r} \tilde{\sigma}_i(\epsilon)\tilde{u}_i(\epsilon)\tilde{v}_i^T(\epsilon). \tag{6}$$

Eventually, we set $\epsilon = 1$.

We consider an asymptotic setting where the number of genes m is fixed, and the number of cells n grows to infinity. For simplicity, we assume the rank r is fixed and a-priori known, so there is no need to estimate it. In what follows, we use the superscript (n) to emphasize this dependence on n when it is relevant (e.g. as $X^{(n)}$ or $u_i^{(n)}$). For future use, we denote the SVD of X by

$$X = \sum_{i=1}^{r} \sigma_i u_i v_i^T,$$

where u_i, v_i are the left and right singular vectors and σ_i are their corresponding singular values. We complete these r vectors to a basis or $\mathbb{R}^n$ by defining u_i for $i = r+1, ..., m$ to be any set of mutually orthogonal vectors which are also orthogonal to $u_1, ..., u_r$ (and similarly for v_i from $i = r+1, ..., n$), and let the corresponding $\sigma_i = 0$. Finally, we denote the eigenvalues of $\frac{1}{n}XX^T$by $\lambda_i = \sigma_i^2/n$, for $r = 1, \ldots, n$.

Assumptions. Prior to stating our main theorem, we first describe our assumptions about the matrix X and the corruption matrix E. We remark that similar assumptions are common in the low-rank matrix completion literature.

A1 **Upper bound on expression values.** There exists a constant $C_0 > 0$ such that

$$X_{k,\ell}^{(n)} \leq C_0.$$

This assumption is met in our biological setting as expression values are bounded and do not depend on the number of cells n.

A2 **Rank r with bounded eigenvalues.** The matrix $X^{(n)}$ has rank r and its singular values $\{\sigma_j\}_{j=1}^r$ are all distinct and scale linearly with the number of cells n. Namely, its $m \times m$ (non-centered) covariance or second moment matrix $\frac{1}{n}X^{(n)}\left(X^{(n)}\right)^T$ has r eigenvalues $\lambda_1 > \lambda_2 > \ldots > \lambda_r$ all with multiplicity one and all bounded away from zero, where we defined $\lambda_j = \sigma_j^2/n$. This assumption that the expression matrix is low rank is standard in scRNA-seq analysis, as it underlies the widespread use of principal component analysis as a preprocessing step. Genes do not act independently, but often form gene modules of highly correlated genes, resulting in low-rank structure.

A3 **Small dropout rate.** The dropout rate $0 < p < 1$ is fixed and sufficiently small in comparison to the spectral gaps between eigenvalues of XX^T/n. Namely,

$$\sqrt{p} \ll \frac{1}{C_0}\frac{\lambda_j - \lambda_{j+1}}{\sqrt{\lambda_1}} \quad \text{for } j = 1, \ldots, r. \tag{7}$$

The assumption that p is small is to bound the size of the error, which is standard in matrix estimation literature. We further assume that p is constant to simplify the analysis.

A4 **Delocalization of right singular vectors.** There exists a constant $C_1 > 0$ such that for all singular vectors $v_i \in \mathbb{R}^n$,

$$|v_i^{(n)}(\ell)| \leq C_1/\sqrt{n} \quad \text{for all } \ell = 1, \ldots, n.$$

Given that $\|v_i\| = 1$, this assumption implies that the large entries of v_i are not concentrated on only a small number of coordinates. In the setting of scRNA-seq, this assumption is consistent with the fact that each gene expression pattern appears in at least a fixed fraction of the cells. Namely, there is no singular vector $v_i^{(n)}$ which is supported on a negligibly small number of cells.

A5 **Interaction of X and right singular vectors.** We assume there is a strictly positive constant c_1 such that for any given gene k and any singular vector v_i, the set

$$B_{i,k}^{(n)} = \left\{ \ell \,\middle|\, |X_{k\ell}^{(n)} v_{i\ell}| \geq c_1/\sqrt{n} \right\}.$$

has non-negligible size with respect to the total number of cells n. Namely, $|B_{i,k}^{(n)}|/n \geq \rho > 0$. This condition is consistent with our assumption that, as $n \to \infty$, a gene cannot be expressed in only a finite number of cells.

Now, we can state our main result.

Theorem 1. *Let $\tilde{X}$ be an $m \times n$ matrix of the form (1) with error matrix E as in Eq. (2). Suppose that Assumptions A1-A5 all hold. Then,*

(i) to leading order in ϵ, the expected value of the rank-r matrix $\tilde{Z}(\epsilon)$ of Eq. (6) is equal to the original matrix X shrunk towards zero,

$$\mathbb{E}[\tilde{Z}(\epsilon)] = (1-p)X + O\left(p\epsilon^2\right).$$

(ii) to leading order in ϵ, as $n \to \infty$, the distribution of $\tilde{Z}_{ij}(\epsilon) - \mathbb{E}[\tilde{Z}_{ij}(\epsilon)]$ is asymptotically Gaussian.

Remark 2. By Theorem 1, if $X_{ij} = 0$, up to an error term of $O(p)$, the distribution of $\tilde{Z}_{ij}$ is symmetric around zero. We note that this property is necessary but not sufficient to justify the ALRA algorithm. For example, consider an extreme case where for a gene i, let $J_i = \{j \mid X_{ij} = 0\}$,

and suppose that all the random variables Z_{ij} for $j \in J_i$ are *perfectly* correlated. Then, the distribution of elements corresponding to true zeros in that row of the matrix would not be symmetric around zero; rather, it would be a single number. To fully justify ALRA, it suffices to show that most pairs of random variables in each row i are weakly correlated. That is, one must bound the covariance between the jth and kth elements of the ith row of Z,

$$\mathbb{E}[(\tilde{Z}_{ij} - \mathbb{E}[\tilde{Z}_{ij}])(\tilde{Z}_{ik} - \mathbb{E}[\tilde{Z}_{ik}])].$$

Providing an analytical bound for these higher order terms is difficult. However, we later show by means of simulation that in practical settings, the correlations between different entries in each row of $\tilde{Z}$ are indeed small.

Multinomial model. As stated, Theorem 1 holds under the fixed dropout rate model of Eq. (2). In reality, the probability to observe $\tilde{X}_{ij} = 0$ may depend on the true value X_{ij} and thus not be uniform over all entries (i, j). A widely-accepted model for scRNA-seq is that each cell is sampled from a multinomial distribution parameterized by the relative abundance of each gene and the number of transcripts in that cell. Specifically, as in [9], let Π be an $m \times n$ matrix where $\Pi_{ij} \geq 0$ denotes the (unknown) relative abundance of transcripts from gene i in cell j, so that by definition, $\sum_{i=1}^{m} \Pi_{ij} = 1$. The $j-$th column (cell) of the measured matrix $\tilde{Y}$ is modeled as

$$\tilde{Y}_{:,j} \sim \text{Multinomial}(\Pi_{:,j}, N_j), \tag{8}$$

where N_j is the total number of transcripts measured in the j-th cell. Since the number of transcripts measured N_j is much smaller than the true number of transcripts, many genes are not sampled, resulting in zero values we wish to impute. For simplicity of presentation, we assume that $N_j = N$ for some N, and we denote $\tilde{X} = \tilde{Y}/N$. Note that as $N \to \infty$, $\tilde{X} \to \Pi$.

Under this model, given the normalized matrix $\tilde{X}$, the task is to estimate the abundance matrix Π. As before, we assume that the true abundance matrix Π is rank-r. To present an analogue of Theorem 1 for this setting, let the true unobserved matrix $X = \Pi$ and define the error matrix $\tilde{E} = \tilde{X} - \Pi = \frac{\tilde{Y}}{N} - \Pi$. Note that the variance of an entry in the observed matrix is given by the following expression,

$$Var\left(\tilde{E}_{ij}\right) = Var\left(\frac{\tilde{Y}_{ij}}{N} - \Pi_{ij}\right) = \frac{\Pi_{ij}(1 - \Pi_{ij})}{N} \leq \frac{1}{N}.$$

Hence, the term $p(1-p)$ where p was the drop-out probability is replaced by the term $1/N$. Similarly, Assumption A3 which limited how large can the dropout probability be, has to be replaced by the following condition, that states that the total number of transcript reads in each cell is sufficiently large. Specifically, Eq. (7) is replaced by

$$\frac{1}{\sqrt{N}} \ll \frac{\lambda_j - \lambda_{j+1}}{\sqrt{\lambda_1}} \quad \text{for } j = 1, \ldots, r.$$

As in Theorem 1, we introduce the one-dimensional family of matrices,

$$\tilde{X}(\epsilon) = \Pi + \epsilon \tilde{E},$$

and define $\tilde{Z}(\epsilon)$ as the rank-r SVD of the matrix $\tilde{X}(\epsilon)$.

The final issue before applying Theorem 1 is to note that the matrix $\tilde{E}$, although it has mean zero, its entries are not independent, but rather weakly dependent, since for each column they sum to zero. Thus, the spectral norm of $\tilde{E}$ cannot be bounded by Lemma 2 below. Instead, we can use Lemma 3, which provides similar results. Namely, $\|\tilde{E}\|$ is tightly concentrated around its mean, and its mean is bounded by $\sqrt{2n}/\sqrt{N} + c\sqrt{\log(n+m)}$ for suitable constant c.

Another important point to make is that the entries $\tilde{E}_{ij}$ in different columns are independent, as these correspond to different cells. Therefore, the Lyapunov condition stated in Lemma 1 below, critical to prove asymptotic Gaussianity, can still be used verbatim, as it only considers sums across columns (cells) for a fixed row (gene).

The proof of Theorem 1 does not use anything else about the matrix $\tilde{E}$ until Eq. 23, where we show that $\mathbb{E}\left[u_i^T \tilde{E} v_j\right] = 0$. But this is also true in the multinomial setting, since

$$\mathbb{E}\left[u_i^T \tilde{E} v_i\right] = \mathbb{E}\left[u_i^T \frac{\tilde{Y}}{N} v_i\right] - u_i^T \Pi v_i = 0.$$

Therefore, under the multinomial model, Theorem 1 continues to hold, namely

$$\mathbb{E}[\tilde{Z}(\epsilon)] = \Pi + O\left(\epsilon^2\right),$$

and the distribution of $\tilde{Z}_{ij}(\epsilon) - \mathbb{E}[\tilde{Z}_{ij}(\epsilon)]$ is asymptotically Gaussian.

Variance Stabilization. An important feature of distributions such as the multinomial, Poisson, or negative binomial—which are commonly used to model scRNA-seq data—is that the variance depends on the mean. Highly expressed genes (such as housekeeping genes, for example) thus have the highest variance, even if they are not differentially expressed between cell types. This variance dominates the top principal components of the dataset, displacing biological variation of interest deeper into the spectrum. Given the relationship between the mean and variance, the delta method can be used to derive a variance stabilizing transformation (VST), removing the dependence of the variance on the mean. In scRNA-sequencing, by far the most commonly used transformation is the log-transform, which is the VST for a negative binomial distribution [1]. Specifically, expression values are column-scaled (i.e. each cell is divided by the sum of its entries), an "offset" of 1 is added, and the log is taken.

In Figure 22, we demonstrate the importance of variance stabilization using the Hrvatin et al. dataset. With column scaling but not log-normalization, the right singular vectors are dominated by a small number of highly expressed genes. For example, the gene Hbb-bt accounts for over 95% of the mass of v_{10}. In contrast, after log-normalization, the mass of each right singular vector is shared among a much larger number of genes, as it should be if it is to represent a "module" of correlated genes.

Empirically, preservation of biological zeros does not appear to be dependent on variance-stabilization (Supplemental Tables 10-12). However, other downstream analyses like t-SNE and clustering depend strongly on it. For this reason, prior to applying ALRA, we scale the columns to sum to 10,000, add a "pseudo-count" of 1, and apply a log transform. As shown in the main text, the choice of scaling factors other than 10,000 does not affect the preservation of biological zeros. Finally, we note that more recently developed transformations for scRNA-seq may also be effective, but were not evaluated in this study.

3. Proof of Theorem 1

The proof consists of the following steps: First, we derive expressions for the singular values and singular vectors of the perturbed matrix $\tilde{X}(\epsilon)$ in terms of the singular values and singular vectors of the original matrix X and of the perturbation matrix $\tilde{E}$. We then use these expressions to obtain the desired form for the expected value of $\tilde{Z}(\epsilon)$. Finally, we apply a central limit theorem to each of the terms that appear in the expression for $\tilde{Z}(\epsilon)$, hence showing that when n is large, to leading order in ϵ, the distribution of each element $\tilde{Z}_{ij}$ is approximately Gaussian.

3.1. **Auxiliary Lemmas.** Before we proceed with our analysis, we state three auxiliary lemmas that we shall use. The first is a classical central limit theorem for sums of independent though not necessarily identically distributed random variables. It states a sufficient condition for their sum to converge to a Gaussian distribution. For its proof, see for example [7, Theorem 2.7.2]. The second concerns the norm of a random rectangular matrix with independent entries. For sake of completeness, we present a proof of this result, which essentially follows from [2]. The third lemma concerns the norm of a random rectangular matrix with independent columns, which are the deviations of a multinomial distribution from its mean. Its proof, which we also detail for the sake of completeness, follows from [10].

Lemma 1 (Lyapunov Condition for Triangular Arrays). *For $n = 1, 2, \dots,$ and $\ell = 1, \dots, n$, let $Y_{n\ell}$ be an infinite triangular array of random variables, with finite mean and variance, $\mathbb{E}[Y_{n\ell}] = \mu_{n\ell}$, and $Var(Y_{n\ell}) = \sigma^2_{n\ell} < \infty$. Assume that for each $k \in \mathbb{N}$, the random variables $Y_{k1}, ..., Y_{kk}$ are independent. Define $s_n^2 = \sum_{\ell=1}^n \sigma^2_{n\ell}$, and suppose that*

$$\lim_{n\to\infty} \frac{\left[\sum_{\ell=1}^n \mathbb{E}\left|Y_{n\ell} - \mu_{n\ell}\right|^3\right]^2}{(s_n^2)^3} = 0. \tag{9}$$

Then, the sum $\sum_{\ell=1}^n (Y_{n\ell} - \mu_{n\ell})/s_n$ converges to a $N(0,1)$ distribution as $n \to \infty$.

Lemma 2. *Let X be an $m \times n$ matrix with $n \geq m$ that satisfies assumption A1 above, and let $\tilde{E}$ be the matrix given by Eq. (4). Then, the spectral norm of $\tilde{E}$ is tightly concentrated around its mean,*

$$\Pr(\|\tilde{E}\| - \mathbb{E}[\|\tilde{E}\|] > t) \leq e^{-t^2/2C_0^2}. \tag{10}$$

Further, its mean value is bounded by

$$\mathbb{E}[\|\tilde{E}\|] \leq 3C_0\sqrt{p(1-p)}\sqrt{n} + c\, C_0\sqrt{\log(n+m)} \tag{11}$$

where c is a universal constant, independent of m, n and of X.

Proof. First, recall that by Eq. (4), the random matrix $\tilde{E}$ has zero mean and independent entries. Moreover, by assumption A1, all its entries are bounded in an interval of length less than or equal to C_0. Since the largest singular value of a matrix is a convex Lipschitz function, Eq. (10) directly follows from standard concentration results for convex Lipschitz functions, see Theorem 6.10 and example 6.11 in [3].

To analyze the mean of its spectral norm, we use recent results regarding the norm of random symmetric matrices whose entries are independent and symmetrically distributed random variables, see Corollaries 3.6 and 3.12 in [2]. To this end, rather than considering the rectangular matrix $\tilde{E}$, we instead study the symmetric matrix A of size $(n+m) \times (n+m)$, given by

$$A = \begin{pmatrix} 0 & \tilde{E} \\ \tilde{E}^T & 0 \end{pmatrix}.$$

It is easy to verify that $\|A\| = \|\tilde{E}\|$. However, its entries are not symmetrically distributed, as required by the above mentioned corollaries. Next, we thus apply a standard symmetrization trick. Let A' an independent copy of A, and let $W = A - A'$. By Jensen's inequality,

$$\mathbb{E}[\|A\|] = \mathbb{E}[\|A - \mathbb{E}[A']\|] \leq \mathbb{E}[\|A - A'\|] = \mathbb{E}[\|W\|].$$

By construction, W is symmetric and the distribution law of each of its entries W_{ij} is symmetric. Furthermore, its upper diagonal entries are independent with mean zero and variance given by

$$Var(W_{ij}) = Var(A_{ij} - A'_{ij}) = 2Var(A_{ij}).$$

By Eq. (5), these may be bounded as follows

$$Var(W_{ij}) \leq 2p(1-p)C_0^2.$$

Next, following [2, page 2497], we define the following two quantities,

$$\tilde{\sigma} = \max_i \sqrt{\sum_j \mathbb{E}[W_{ij}^2]} \leq C_0\sqrt{2p(1-p)}\sqrt{n}$$

and

$$\sigma_{\max} = \max_{i,j} |W_{ij}| \leq 2C_0.$$

Then, by Corollary 3.6 in [2], see also the second equation in page 2497 of their paper, for any $0 < \epsilon < 1/2$, there exist a suitable constant C_ϵ such that

$$\mathbb{E}[\|W\|] \leq (1+\epsilon)2\tilde{\sigma} + C_\epsilon \sigma_{\max}\sqrt{\log(n+m)}.$$

Taking $\epsilon = 1/2$ and the bounds on $\tilde{\sigma}$ and $\sigma_{\max}$ yields Eq. (11). $\square$

Lemma 3. *Let Π be an $m \times n$ matrix of n probability vectors. Let $\tilde{Y}$ be a matrix whose j-columns are multinomial with total count N and with probability vector $\Pi_{:,j}$, as in Eq. (8). Consider the matrix $\tilde{E} = \tilde{Y}/N - \Pi$. Then, for some universal constant C,*

$$\mathbb{E}\left[\|\tilde{E}\|\right] \leq \sqrt{\frac{2}{N}}\sqrt{n} + C\sqrt{\log(\min(m,n))}. \tag{12}$$

In addition, the spectral norm of $\tilde{E}$ is tightly concentrated around its mean.

Proof. First, note that by the Cauchy-Schwarz inequality, $\mathbb{E}[\|\tilde{E}\|] \leq (\mathbb{E}[\|\tilde{E}\|^2])^{1/2}$. To bound the latter, we consider the $n \times m$ matrix A whose j-th row is the column $\tilde{E}_{:,j}$. By definition, the rows of A are independent. We may thus invoke Theorem 5.48 in [10]. The quantity Σ appearing in Theorem 5.48 is given by $\Sigma = \frac{1}{n}\sum \Sigma_j$ where $\Sigma_j = \mathbb{E}[A_j \otimes A_j]$, see Remark 5.49 in [10]. Note that since the j-th row of A, namely $\tilde{E}_{:,j}$ is the difference between two probability vectors,

$$\|A_j\|_2 = \|\tilde{E}_{:,j}\|_2 \leq \|\tilde{Y}_{:,j}/N\|_2 + \|\Pi_{:,j}\|_2 \leq 2.$$

Next, note that

$$\Sigma_j = \mathbb{E}[A_j \otimes A_j] = \mathbb{E}[E_{:,j}E_{:,j}^T] = \frac{1}{N}\left[diag(\Pi_{:,j}) - \Pi_{:,j}\Pi_{:,j}^T\right]$$

Hence, $\|\Sigma_j\| \leq 2/N$, and similarly $\|\Sigma\| = \|\frac{1}{n}\sum \Sigma_j\| \leq 2/N$ as well. Eq. (12) and the tight concentration now follow from Theorem 5.48. □

3.2. **Perturbed eigenvalues.** We study the left singular vectors $\tilde{u}_i(\epsilon)$ by a perturbation analysis, similar to [8] and [4]. To this end, we analyze the following symmetric $m \times m$ matrix $G(\epsilon)$, which is a smoothly differentiable function of ϵ,

$$\begin{aligned} G(\epsilon) &= \frac{1}{n}\tilde{X}(\epsilon)\tilde{X}(\epsilon)^T = \left[(1-p)X + \epsilon\tilde{E}\right]\left[(1-p)X + \epsilon\tilde{E}\right]^T \\ &= \underbrace{(1-p)^2\frac{1}{n}XX^T}_{G_0} + \epsilon\underbrace{\frac{(1-p)}{n}\left(X\tilde{E}^T + \tilde{E}X^T\right)}_{G_1} + \epsilon^2\underbrace{\frac{1}{n}\left(\tilde{E}\tilde{E}^T\right)}_{G_2} \\ &= G_0 + \epsilon G_1 + \epsilon^2 G_2. \end{aligned} \tag{13}$$

Before we expand the eigenvalues and eigenvectors in ϵ, it is instructive to consider the norms of the perturbation matrices G_1 and G_2 as compared to those of the base matrix G_0. First, recall that by assumption A2, the matrix G_0 is of rank r and its non-zero eigenvalues are equal to $\lambda_j(G_0) = (1-p)^2\lambda_j$, for $j = 1, \ldots, r$. By assumption A2, the smallest eigenvalue is bounded away from zero. Next, consider the matrix $G_2 = \frac{1}{n}\tilde{E}\tilde{E}^T$. By Lemma 2, Eq. (10), since $\|\tilde{E}\|$ is tightly concentrated around its mean, then $\|\tilde{E}\|^2$ is tightly concentrated around its mean as well, and furthermore, $\mathbb{E}[\|\tilde{E}\|^2] \leq C\left[\mathbb{E}[\|\tilde{E}\|]\right]^2$ for a suitable constant C. Combining this with Eq. (11) of Lemma 2 gives

$$\begin{aligned} \mathbb{E}[\|G_2\|] &\leq \frac{C}{n}\left(3C_0\sqrt{p(1-p)}\sqrt{n} + cC_0\sqrt{\log(n+m)}\right)^2 \\ &\leq 9p(1-p)C_0^2C + 6cC_0^2C\sqrt{p(1-p)}\sqrt{\frac{\log(n+m)}{n}} + c^2C_0^2C\frac{\log(n+m)}{n}. \end{aligned}$$

For any fixed $0 < p < 1$, as n tends to infinity the last two terms on the right hand size above tend to zero. Hence, for $n \gg 1$ these two terms are negligible with respect to the first one. Furthermore, for sufficiently small p, $\mathbb{E}[\|G_2\|] \ll \lambda_r(G_0)$. Hence, even at $\epsilon = 1$, the second order term G_2 is small with respect to even the smallest signal in G_0.

Regarding the first perturbation matrix $G_1 = \frac{1}{n}(1-p)(X\tilde{E}^T + \tilde{E}X^T)$, note that by definition, $\mathbb{E}[G_1] = 0$. As for its expected spectral norm, again by Lemma 2, for $n \gg 1$,

$$\mathbb{E}[\|G_1\|] \leq \frac{2}{n}(1-p)\|X\| \cdot \mathbb{E}[\|\tilde{E}\|] \leq 6(1-p)\sqrt{\lambda_1}C_0\sqrt{p(1-p)}.$$

Note that $\|G_1\|$ scales like $\sqrt{p}$, whereas $\|G_2\|$ scales like p. Thus, for small p, $\|G_1\|$ is much larger than $\|G_2\|$. In our perturbation analysis, we shall thus consider only the first order terms, which as we shall see depend only on G_1.

Next, for the matrix G_1 to have a relatively small effect on the top r eigenvectors of the original low rank matrix G_0, the key requirement is that the eigenvalues $\lambda_j(\epsilon)$ will not cross each other [6]. Since by Weyl's inequality $\lambda_j(G_0 + G_1) \le \lambda_j(G_0) + \|G_1\|$, a sufficient condition to ensure that with high probability, no such crossover occurred is precisely Eq. (7) of assumption A3. We remark that Assumption A3 may be weakened to less stringent conditions, see for example [5].

We now proceed to expand the eigenvalues and eigenvectors of $G(\epsilon)$ in ϵ. Since $G(\epsilon)$ is symmetric and quadratic in ϵ, for a fixed X and $\tilde{E}$, for sufficiently small ϵ, the r leading eigenvalues and eigenvectors of $G(\epsilon)$ are all analytic functions of ϵ (see, e.g., Chapter 2, Theorem 6.1 of [6]). Specifically, for $k = 1, \dots, r$ we may expand the eigenpair $(\tilde{u}_k, \tilde{\lambda}_k)$ in a Taylor series,

$$\tilde{u}_k(\epsilon) = u_k + \epsilon \tilde{u}_k^{(1)} + \epsilon^2 \tilde{u}_k^{(2)} + \dots \tag{14}$$

$$\tilde{\lambda}_k(\epsilon) = \lambda_k(G_0) + \epsilon \tilde{\lambda}_k^{(1)} + \epsilon^2 \tilde{\lambda}_k^{(2)} + \dots \tag{15}$$

Assumption A3 together with the tight concentration of $\|\tilde{E}\|$ around its mean, imply that with high probability, no cross-over of eigenvalues has occurred and thus the above expansions are valid up to $\epsilon = 1$. Since eigenvectors are only defined up to a multiplicative normalization factor, the form of Eq. (14) implies that the chosen normalization is such that $\tilde{u}_k^{(1)}$ and all other higher order perturbations are orthogonal to u_k.

Inserting Eqs. (13), (14), and (15) into the characteristic equation $G(\epsilon)\tilde{u}_k(\epsilon) = \tilde{u}_k(\epsilon)\tilde{\lambda}_k(\epsilon)$ gives

$$(G_0 + \epsilon G_1 + \epsilon^2 G_2)(u_k + \epsilon \tilde{u}_k^{(1)} + \epsilon^2 \tilde{u}_k^{(2)} + \dots) = (u_k + \epsilon \tilde{u}_k^{(1)} + \epsilon^2 \tilde{u}_k^{(2)} + \dots)(\lambda_k(G_0) + \epsilon \tilde{\lambda}_k^{(1)} + \epsilon^2 \tilde{\lambda}_k^{(2)} + \dots).$$

We now equate terms with equal powers of ϵ. The $O(1)$ terms recover the original eigenvalues and eigenvectors of G_0, consistent with the expansion in Eqs. (14)-(15). The $O(\epsilon)$ terms give

$$G_1 u_k + G_0 \tilde{u}_k^{(1)} = \lambda_k(G_0)\tilde{u}_k^{(1)} + \tilde{\lambda}_k^{(1)} u_k. \tag{16}$$

Since $\tilde{u}_k^{(1)}$ is orthogonal to u_k and $u_k^T u_k = 1$, multiplying the above equation by u_k^T gives

$$\tilde{\lambda}_k^{(1)} = u_k^T G_1 u_k.$$

Inserting the expression for G_1, and recalling that $X^T u_k = \sigma_k v_k = \sqrt{n\lambda_k} v_k$, yields the following closed form expression for the leading order perturbation of λ_k,

$$\tilde{\lambda}_k^{(1)} = \frac{1-p}{n} u_k^T (X\tilde{E}^T + \tilde{E}X^T) u_k = \frac{2(1-p)}{n} u_k^T \tilde{E} X^T u_k = \frac{2(1-p)}{\sqrt{n}} \sqrt{\lambda_k} \left(u_k^T \tilde{E} v_k \right). \tag{17}$$

In other words, the leading order term in the perturbation of λ_k is determined by the interaction of left and right singular vectors with the error matrix $\tilde{E}$.

3.3. **Perturbed eigenvectors.** Next we study the effect of $\tilde{E}$ on the leading eigenvectors. We rearrange Eq. (16), and consider the k-th eigenvector,

$$(G_0 - \lambda_k(G_0)I)\tilde{u}_k^{(1)} = \tilde{\lambda}_k^{(1)} u_k - G_1 u_k. \tag{18}$$

We expand the first order perturbation in the singular vectors of G_0,

$$\tilde{u}_k^{(1)} = \sum_{i=1}^{m} a_i u_i, \tag{19}$$

where $a_i = u_i^T \tilde{u}_k^{(1)}$. Inserting Eq. (19) into Eq. (18) gives

$$(1-p)^2 \sum_{\substack{i=1 \\ i \neq k}} (\lambda_i - \lambda_k) a_i u_i = \tilde{\lambda}_k^{(1)} u_k - G_1 u_k.$$

Since $u_k^{(i)}$ are orthogonal to u_k, $a_k = 0$. To obtain the other coefficients a_i, we multiply both sides by u_i^T, and plug in the definition of G_1

$$a_i = \frac{1}{(1-p)^2}\frac{u_i^T G_1 u_k}{\lambda_k - \lambda_i} = \frac{1}{\lambda_k - \lambda_i}\frac{1}{n(1-p)}\left(u_i^T X \tilde{E}^T u_k + u_i^T \tilde{E} X^T u_k\right).$$

Given that $u_i^T X = \sigma_i v_i^T = \sqrt{n\lambda_i} v_i^T$ yields that

$$a_i = \frac{1}{\sqrt{n}}\frac{1}{1-p}\left[\frac{\sqrt{\lambda_i}}{\lambda_k - \lambda_i} v_i^T \tilde{E}^T u_k + \frac{\sqrt{\lambda_k}}{\lambda_k - \lambda_i} u_i^T \tilde{E} v_k\right].$$

Inserting the above into Eq. (19) gives the following expression for the first order perturbation of the k-th eigenvector,

$$\tilde{u}_k^{(1)} = \frac{1}{\sqrt{n}}\frac{1}{1-p}\sum_{\substack{i=1\\ i\neq k}}^{m} \frac{1}{\lambda_k - \lambda_i}(\sqrt{\lambda_i} u_k^T \tilde{E} v_i + \sqrt{\lambda_k} u_i^T \tilde{E} v_k) u_i. \tag{20}$$

The analysis proceeds similarly for the first order perturbation of the right singular vector v_k. Since by definition, v_k is the k-th eigenvector of $\frac{1}{n}\tilde{X}^T\tilde{X}$, the analogue of Eq. (20) is

$$\tilde{v}_k^{(1)} = \frac{1}{\sqrt{n}}\frac{1}{1-p}\sum_{\substack{i=1\\ i\neq k}}^{n} \frac{1}{\lambda_k - \lambda_i}(\sqrt{\lambda_i} u_i^T \tilde{E} v_k + \sqrt{\lambda_k} u_k^T \tilde{E} v_i) v_i. \tag{21}$$

3.4. **Rank-r approximation to $\tilde{X}$.** Recall that our object of interest is the best rank-r approximation to the perturbed matrix $\tilde{X}$, which we called $\tilde{Z}$,

$$\tilde{Z} = \sqrt{n}\sum_{i=1}^{r}\sqrt{\tilde{\lambda}_i}\tilde{u}_i\tilde{v}_i^T.$$

Inserting the Taylor expansions for the eigenvalues and singular vectors gives

$$\begin{aligned}
\tilde{Z}(\epsilon) &= \sqrt{n}\sum_{i=1}^{r}\sqrt{\lambda_i(G_0) + \epsilon\tilde{\lambda}_i^{(1)} + O(\epsilon^2)}\left(u_i + \epsilon\tilde{u}_i^{(1)} + O(\epsilon^2)\right)\left(v_i + \epsilon\tilde{v}_i^{(1)} + O(\epsilon^2)\right)^T \\
&= \sqrt{n}\sum_{i=1}^{r}\sqrt{\lambda_i(G_0)}\left(1 + \epsilon\frac{\tilde{\lambda}_i^{(1)}}{2\lambda_i(G_0)} + O(\epsilon^2)\right)\left(u_i v_i^T + \epsilon(u_i(\tilde{v}_i^{(1)})^T + \tilde{u}_i^{(1)} v_i^T) + O(\epsilon^2)\right) \\
&= \sqrt{n}\sum_{i=1}^{r}\sqrt{\lambda_i(G_0)}u_i v_i^T + \epsilon\sqrt{n}\sum_{i=1}^{r}\left(\frac{\tilde{\lambda}_i^{(1)}}{2\sqrt{\lambda_i(G_0)}}u_i v_i^T + \sqrt{\lambda_i(G_0)}\tilde{u}_i^{(1)}v_i^T + \sqrt{\lambda_i(G_0)}u_i(\tilde{v}_i^{(1)})^T\right) + O(\epsilon^2) \\
&= (1-p)X + \epsilon\sqrt{n}\sum_{i=1}^{r}\left(\frac{\tilde{\lambda}_i^{(1)}}{2\sqrt{\lambda_i(G_0)}}u_i v_i^T + \sqrt{\lambda_i(G_0)}\tilde{u}_i^{(1)}v_i^T + \sqrt{\lambda_i(G_0)}u_i(\tilde{v}_i^{(1)})^T\right) + O(\epsilon^2) \\
&= (1-p)X + \epsilon\tilde{Z}^{(1)} + O(\epsilon^2)
\end{aligned} \tag{22}$$

where $\tilde{Z}^{(1)}$ is the first order perturbation of $\tilde{Z}(\epsilon)$. Plugging in Eqs. (17), (20) and (21),

$$\begin{aligned}
\tilde{Z}^{(1)} =&(1-p)\sum_{i=1}^{r}\left(u_i^T\tilde{E}v_i\right)u_i v_i^T + \\
&\frac{1}{1-p}\sum_{i=1}^{r}\sum_{\substack{j=1\\ j\neq i}}^{m}\frac{\sqrt{\lambda_i}}{\lambda_i - \lambda_j}(\sqrt{\lambda_j}u_i^T\tilde{E}v_j + \sqrt{\lambda_i}u_j^T\tilde{E}v_i)u_j v_i^T + \\
&\frac{1}{1-p}\sum_{i=1}^{r}\sum_{\substack{j=1\\ j\neq i}}^{n}\frac{\sqrt{\lambda_i}}{\lambda_i - \lambda_j}(\sqrt{\lambda_j}u_j^T\tilde{E}v_i + \sqrt{\lambda_i}u_i^T\tilde{E}v_j)u_i v_j^T
\end{aligned} \tag{23}$$

The first part of Theorem 1 follows by showing that the expected value of this first order perturbation is zero. Indeed, since $\tilde{Z}^{(1)}$ is linear in the entries of $\tilde{E}$ and since $\mathbb{E}[\tilde{E}] = 0$, then indeed $\mathbb{E}[\tilde{Z}^{(1)}] = 0$ as well, and part (i) follows.

To prove the second part of the Theorem we shall use Lemma 1. The term that shows up repeatedly in the expression (23) for $\tilde{Z}^{(1)}$ is

$$u_j^T \tilde{E} v_i = \sum_{k=1}^{m} \sum_{\ell=1}^{n} u_j(k) \tilde{E}(k,\ell) v_i(\ell) = \sum_{k=1}^{m} u_j(k) \sum_{\ell=1}^{n} \tilde{E}(k,\ell) v_i(\ell).$$

To prove that $\tilde{Z}^{(1)}$ is asymptotically Gaussian, it suffices to show that each of the individual sums $\sum_{\ell=1}^{n} \tilde{E}^{(n)}(k,\ell) v_i^{(n)}(\ell)$ is asymptotically Gaussian as $n \to \infty$. Intuitively, for this to hold we need on the one hand many of the coefficients $\tilde{E}(k,\ell) v_i^{(n)}(\ell)$ to be non-zero, and in fact bounded away from zero, and on the other hand that the sum is not dominated by very few large coefficients. These two conditions are captured by our two assumptions A4 and A5. Let us now show that under these two assumptions, the Lyapunov condition is satisfied,

$$\lim_{n \to \infty} \frac{\sum_{\ell=1}^{n} \mathbb{E} |\tilde{E}^{(n)}(k,\ell) v_i^{(n)}(\ell)|^3}{\sigma_n^3} \to 0$$

where $\sigma_n^2 = \sum_{\ell=1}^{n} \mathbb{E} \left[\tilde{E}^{(n)}(k,\ell) v_i^{(n)}(\ell) \right]^2$. We begin by upper bounding the numerator. Combining Eq. (4) with assumptions A1 and A4,

$$\begin{aligned} \sum_{\ell=1}^{n} \mathbb{E} \left[|\tilde{E}^{(n)}(k,\ell) v_i^{(n)}(\ell)|^3 \right] &= \sum_{\ell=1}^{n} \left[p(1-p)^3 + (1-p)p^3 \right] \left| X^{(n)}(k,\ell) v_i^{(n)}(\ell) \right|^3 \\ &\leq p(1-p)^2 n C_0^3 \frac{C_1^3}{n^{3/2}} \leq \frac{C}{\sqrt{n}}. \end{aligned}$$

Next, we lower bound the denominator. Here, by Eq. (5) and assumption A5,

$$\begin{aligned} \sigma_n^2 &= \sum_{\ell=1}^{n} \mathbb{E} \left[\tilde{E}^{(n)}(k,\ell) v_i^{(n)}(\ell) \right]^2 = \sum_{\ell=1}^{n} p(1-p) \left[\tilde{X}^{(n)}(k,\ell) \right]^2 \left[v_i^{(n)}(\ell) \right]^2 \\ &\geq p(1-p) c_1^2 \rho. \end{aligned}$$

Thus, the Lyapunov condition holds, and each of the above terms is asymptotically Gaussian.

Simulation

By Theorem 1, the entries corresponding to biological zeros are asymptotically Gaussian. However, as discussed in Remark 1, to fully justify ALRA, the correlations between elements corresponding to biological zeros in each row of Z needs to be small. We here present simulation results that show that empirically this is indeed the case. Our simulation is based on the multinomial model described above and also in the Online Methods. Specifically, we used bulk RNA-seq samples of purified peripheral blood monocyte (PBMC) populations from ImmGen. Each of the $i = 1, ..., 9$ bulk RNA-seq samples corresponds to a purified cell population. We enumerated the genes as $i = 1, ..., m$, and let n_i^j denote the read counts of gene i in the jth bulk RNA-seq sample. To generate the gene expression profile for the ℓ-th cell $\Pi_{:,\ell}$, we chose one of the 9 cell types with equal probability and denoted the cell type of the ℓ-th cell by $c_\ell \in \{1, ..., 9\}$. Then for each gene i, we let

$$\Pi_{i,\ell} = \frac{n_i^{c_\ell}}{\sum_{k=1}^{m} n_k^{c_\ell}}.$$

In order to set N_ℓ to be a realistic number, we randomly sampled a cell from the purified PBMC dataset (Zheng et al.) and let N_ℓ be the read count in that cell. Before sampling from the multinomial, we filtered the genes and cells of Π (a standard step in many scRNA-seq analyses) based on the mean expression levels over all cells. Specifically, We first removed genes i whose mean expression was less than 25 and then removed cells j whose total number of read counts was

less than 1000. The filtering process resulted in a matrix Π' with slightly fewer number of genes. The filtering process resulted in a matrix Π' with $11,062$ genes and $4,216$ cells.

Finally, we constructed 500 independent realizations of a matrix $\tilde{Y}_{:,\ell}$ from Multinomial$(\Pi'_{:,j}, N_j)$. For a given gene i, we then computed the set of correlations between entries corresponding to biological zeros

$$\rho_i = \{\mathrm{Cor}(Y_{i,j}, Y_{i,j'}) \,|\, \Pi_{i,j} = \Pi_{i,j'} = 0\}\,.$$

Supplementary Figure 23 shows the histogram of the values of these empirical correlations for three randomly chosen genes. As seen from the figure, the correlations are quite small. We remark that the mean correlation is not necessarily zero. For 100 randomly chosen genes, the mean and standard deviation of ρ_i were 0.03 and 0.05, respectively.

Supplementary Tables and Figures

Supplementary Table 1. Re-analyzing developing skins cells from Gupta et al. after imputation. Percentage of cells with > 0 expression for marker genes in wildtype (WT) and mutant (MT).

Marker Genes	**Original**		**ALRA**		**MAGIC**		**SAVER**		**DCA**		**scImpute**	
	WT	MT	WT	MT	WT	MT	WT	MT	WT	MT	WT	MT
Axin2+ & Lef1+	15%	6.4%	47%	22%	100%	100%	77%	76%	100%	100%	77%	58%
Sox2+	2.6%	1.5%	6.9%	4.4%	96%	100%	14%	8.8%	98%	100%	18%	16%

Supplementary Table 2. Ratio of biological zeros preserved (ZP) when imputing purified PBMCs of Stoeckius et al. Ratio of total zeros completed (TC) is also shown.

		**ALRA**		**MAGIC**		**SAVER**		**scImpute**	
	# Genes	ZP	TC	ZP	TC	ZP	TC	ZP	TC
B cells	231	0.93	0.57	0.59	0.85	0.78	0.69	0.99	0.12
Monocytes	297	0.94	0.53	0.38	0.88	0.73	0.70	0.99	0.11
T cells	171	0.92	0.56	0.37	0.92	0.77	0.69	0.99	0.08

Supplementary Table 3. Average number of the k nearest neighbors of a point in ADT space that are also among the k nearest neighbors of the same point in RNA space before and after imputation.

**k**	**Original**	**ALRA**	**MAGIC**	**SAVER**	**DCA**	**scImpute**
50	1.45	3.76	3.99	2.86	4.00	2.19
100	4.89	12.0	12.6	9.33	12.7	7.37
1000	262	420	414	366	426	344

Supplementary Table 4. Ratio of biological zeros preserved (ZP) when imputing purified PBMCs of Stoeckius et al for different settings of k. Ratio of total zeros completed (TC) is also shown.

	**k=15**		**k=17**		**k=19**		**k=21**		**k=23**		**k=25**	
	ZP	TC	ZP	TC	ZP	TC	ZP	TC	ZP	TC	ZP	TC
B cells	0.90	0.64	0.91	0.61	0.92	0.59	0.93	0.58	0.93	0.56	0.94	0.54
Monocytes	0.92	0.60	0.92	0.58	0.93	0.56	0.94	0.54	0.94	0.53	0.94	0.51
T cells	0.88	0.63	0.89	0.61	0.90	0.58	0.91	0.56	0.92	0.55	0.93	0.53

Supplementary Table 5. Markers used for gating experiment

Cell Type	Positive Markers	Negative Markers
T Regulatory cells	CD3G, CD4, IL2RA, CCR4, FOXP3	
Memory CD4 cells	CD3G, CD4, CCR7, SELL	CD8A
Memory CD8 cells	CD3G, CD8A, CCR7, SELL	CD4
Memory B cells	CD27, CD19	CD14, CD3G
Naive B cells	CD19	CD14, CD27, CD3G
Natural Killer cells	NCAM1	CD3G, CD19
Classical Monocytes	CD14	CD3G, CD19, MS4A1, NCAM1, FCGR3A
Intermediate Monocytes	CD14, FCGR3A	CD3G, CD19, MS4A1, NCAM1

Supplementary Table 6. After imputation of the purified PBMCs from Zheng et al., the ratio of biological zeros preserved (ZP) is shown alongside the ratio of total zeros completed, with varying scaling factors used for normalization. The expression values were normalized as $\log(\frac{\alpha}{s_j} \cdot x_{ij} + 1)$, where α is the scaling factor and s_j is the library size for the jth cell. The rank estimated by ALRA is shown as k. With the default scaling factor of 10,000, the results in Figure 2B are obtained.

**Scaling Factor (α)**<br>Rank	**1,000**<br>k=26		**5,000**<br>k=28		**10,000**<br>k=26		**20,000**<br>k=24	
	ZP	TC	ZP	TC	ZP	TC	ZP	TC
B cells	0.90	0.72	0.90	0.71	0.90	0.71	0.90	0.71
CD14+ Monocytes	0.86	0.66	0.87	0.66	0.86	0.66	0.86	0.65
T cells	0.87	0.71	0.87	0.70	0.87	0.70	0.87	0.70
CD56+ NK	0.78	0.74	0.78	0.74	0.78	0.74	0.77	0.74

Supplementary Table 7. After imputation of scRNA-seq data from purified PBMCs of Stoeckius et al., the ratio of biological zeros preserved (ZP) is shown alongside the ratio of total zeros completed (TC), with varying scaling factors used for normalization. The expression values were normalized as $\log(\frac{\alpha}{s_j} \cdot x_{ij} + 1)$, where α is the scaling factor and s_j is the library size for the jth cell. The rank estimated by ALRA is shown as k. With the default scaling factor of 10,000, the results in Supplemental Table 2 are obtained.

**Scaling Factor (α)**<br>Rank	**1,000**<br>k=23		**5,000**<br>k=22		**10,000**<br>k=22		**20,000**<br>k=20	
	ZP	TC	ZP	TC	ZP	TC	ZP	TC
B cells	0.94	0.56	0.93	0.57	0.93	0.57	0.92	0.58
Monocytes	0.94	0.53	0.94	0.54	0.94	0.53	0.93	0.55
T cells	0.92	0.55	0.92	0.56	0.92	0.56	0.91	0.57

Supplementary Table 8. After imputation of a simulated scRNA-seq dataset from Suppelemtntary Figure 2D, the ratio of biological zeros preserved (ZP) is shown alongside the technical zeros completed (TC), with varying scaling factors used for normalization. The expression values were normalized as $\log(\frac{\alpha}{s_j} \cdot x_{ij} + 1)$, where α is the scaling factor and s_j is the library size for the jth cell. The rank estimated by ALRA is shown as k. Scaling factor with 10,000 corresponds to Supplemental Figure 2D.

**Scaling Factor (α)**	**1,000**	**5,000**	**10,000**	**20,000**
Rank (k)	6	7	7	8
Biological zeros preserved	0.99	0.99	0.98	0.99
Technical zeros completed	0.72	0.76	0.76	0.73

Supplementary Table 9. After imputation of the human bronchial epithelial cells, the number of cells expressing SARS-CoV-2 entry genes ACE2, TMPRSS2, and FURIN, varying scaling factors used for normalization. The expression values were normalized as $\log(\frac{\alpha}{s_j} \cdot x_{ij} + 1)$, where α is the scaling factor and s_j is the library size for the jth cell. The rank estimated by ALRA is shown as k.

**Scaling Factor**	**k**	**# triple positive**
1,000	49	1,246
5,000	60	1,080
10,000	62	1,181
20,000	66	1,167

Supplementary Table 10. Effect of log-transform on proportions of biological zeros preserved (ZP) and total zeros completed (TC) in the purified PBMC dataset. “With log” results are reproduced here from Figure 2C.

	With Log		Without Log	
	ZP	TC	ZP	TC
B Cells	0.90	0.71	0.88	0.73
CD14+ Monocytes	0.86	0.66	0.81	0.68
T Cells	0.87	0.70	0.84	0.73
CD56+ NK	0.78	0.74	0.77	0.74

Supplementary Table 11. Effect of log-transform on proportions of biological zeros preserved (ZP) and total zeros completed (TC) in the Stoeckius et al. dataset. “With log” results are reproduced here from Supplemental Table 2.

	With Log		Without Log	
	ZP	TC	ZP	TC
B Cells	0.93	0.57	0.96	0.46
Monocytes	0.94	0.53	0.96	0.42
T Cells	0.92	0.56	0.95	0.45

Supplementary Table 12. Effect of log-transform on proportions of biological zeros preserved (ZP) and technical zeros completed (TC) in the simulated dataset. "With log" results correspond to Figure 2D.

With Log		Without Log	
ZP	TC	ZP	TC
1.00	0.43	0.98	0.76

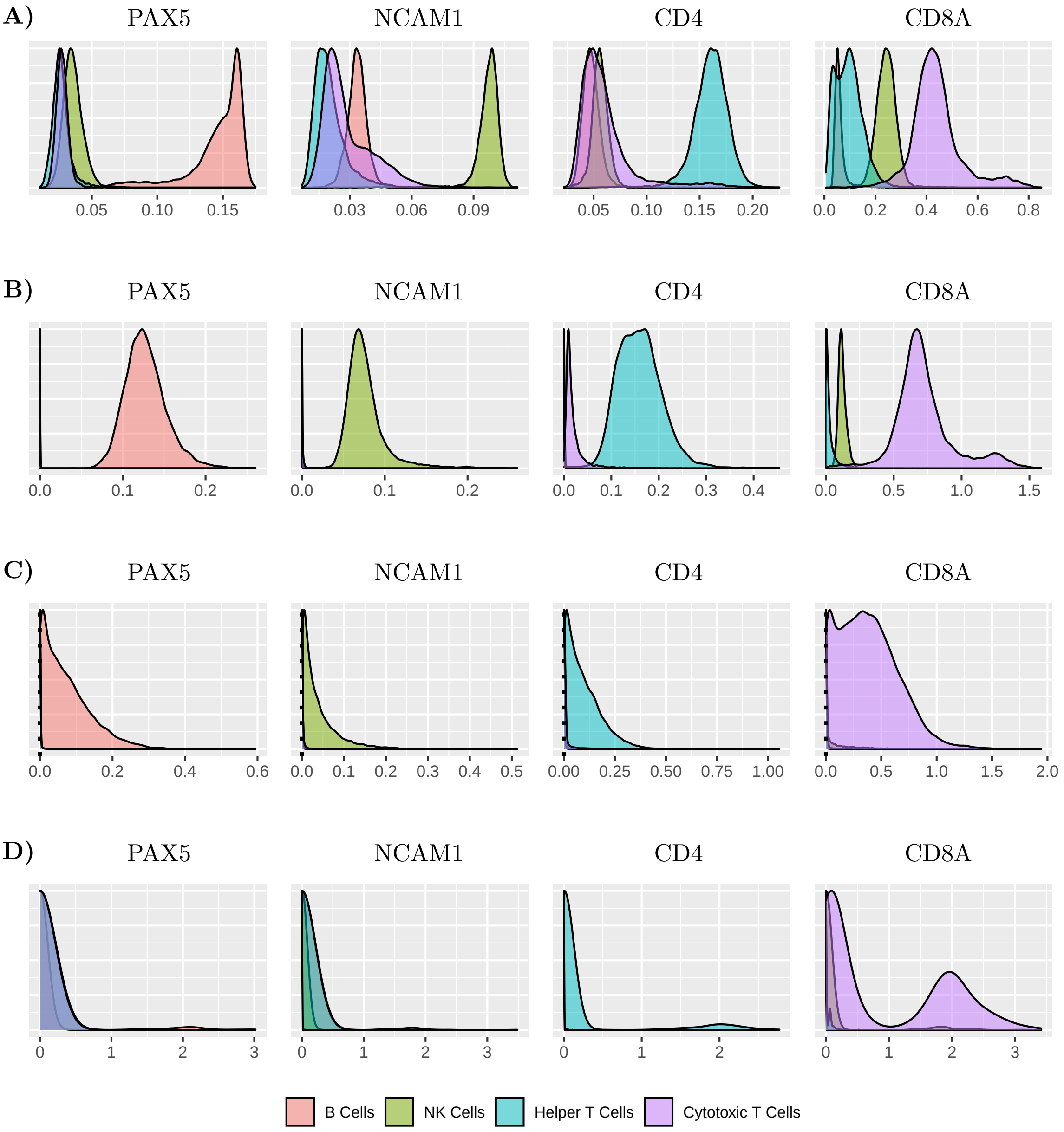

Supplementary Figure 1. Distribution of gene markers PAX5, NCAM1, CD4 and CD8A after imputation of purified PBMCs, colored based on different cell types. A) DCA's output B) MAGIC's output. C) SAVER's output D) scImpute's output

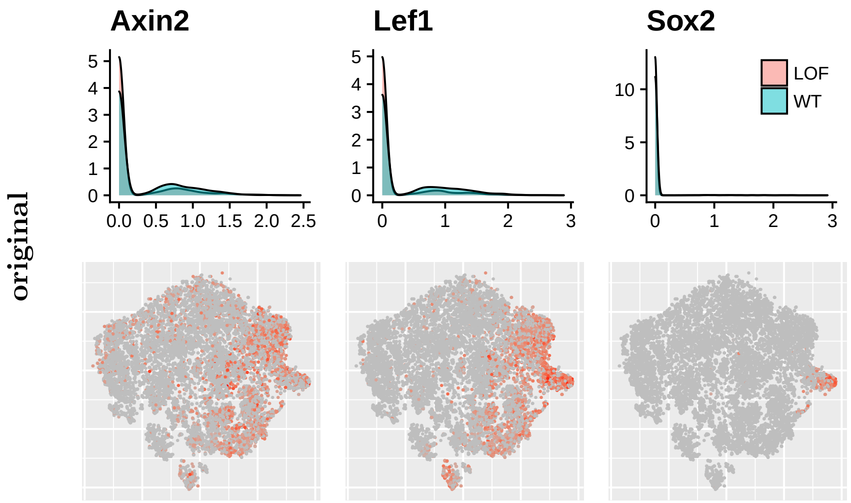

Supplementary Figure 2. Density plots and t-SNE of developing skins cells from Gupta et al, prior to imputation.

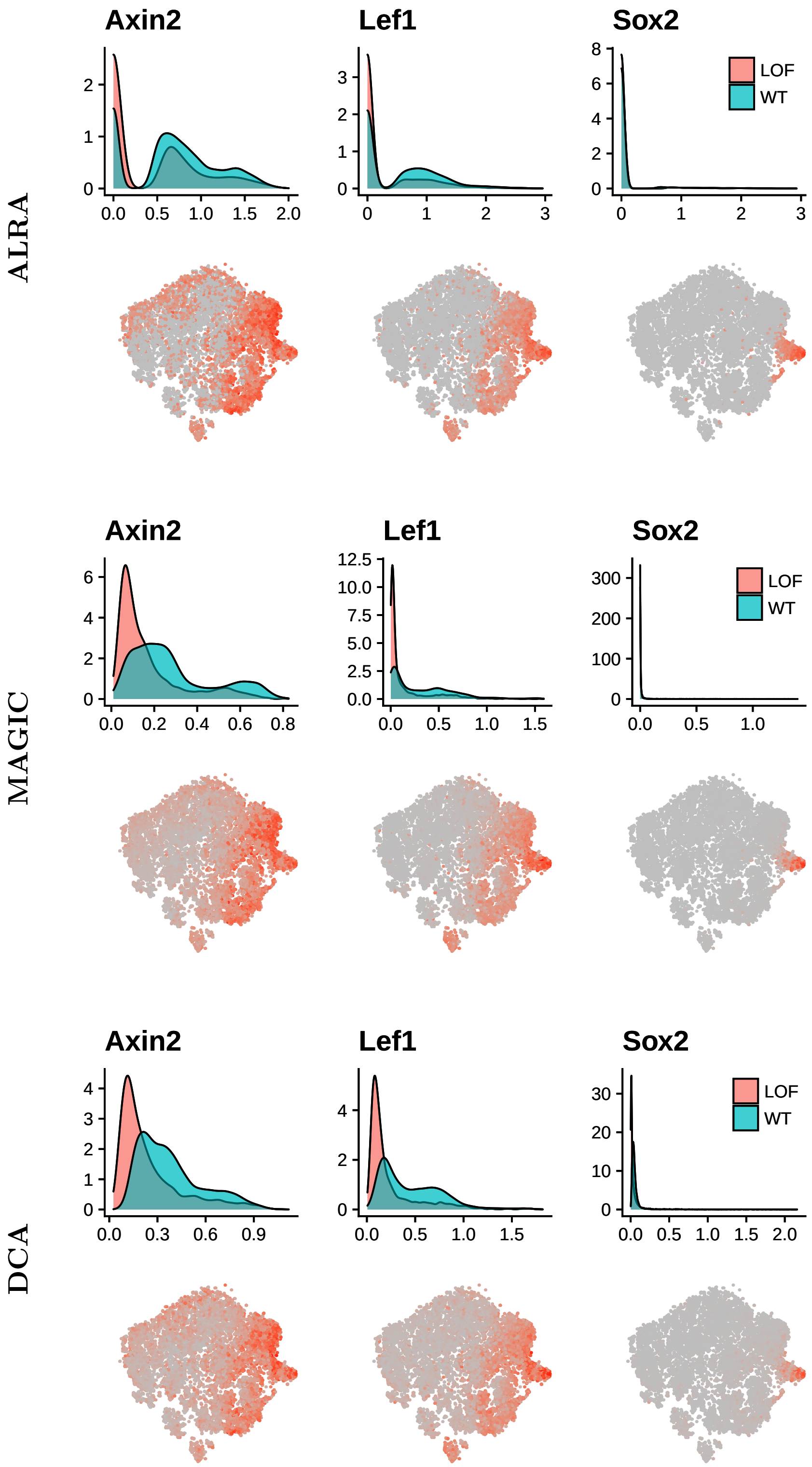

Supplementary Figure 3. Density plots and t-SNE of developing skins cells from Gupta et al. DCA and MAGIC do not preserve biological zeros, and it is not clear how to threshold the values to restore the zeros.

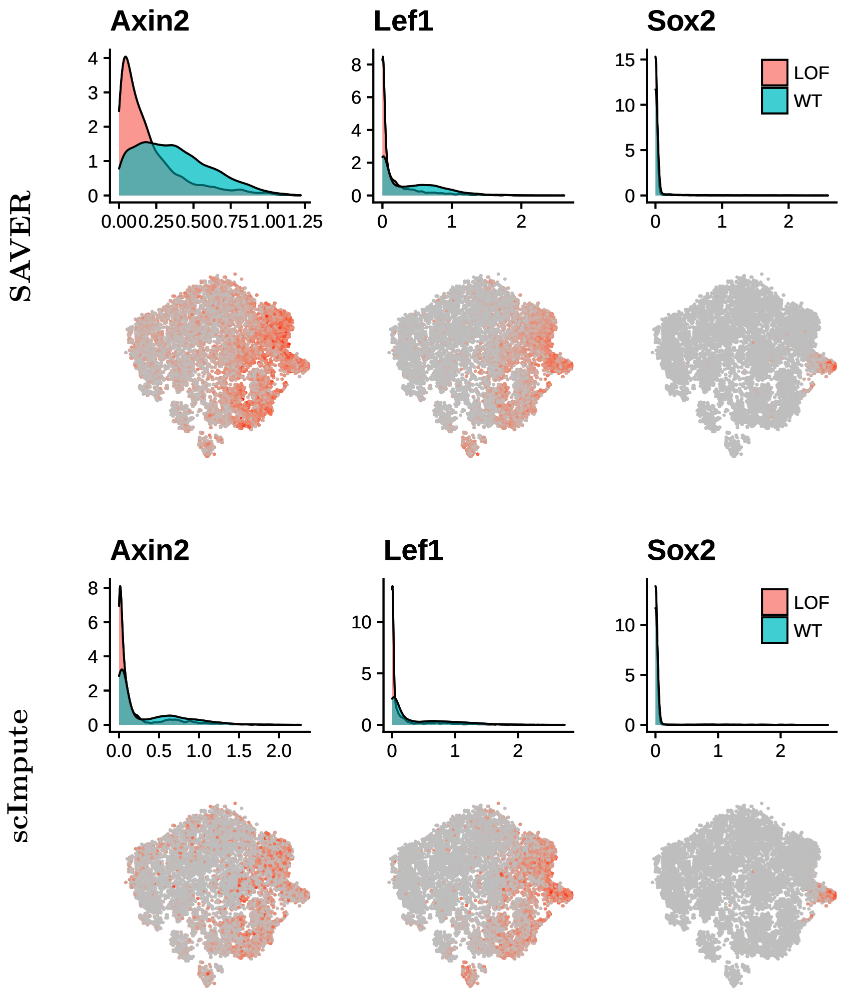

Supplementary Figure 3. (Continued from previous page) Density plots and t-SNE of developing skins cells from Gupta et al.

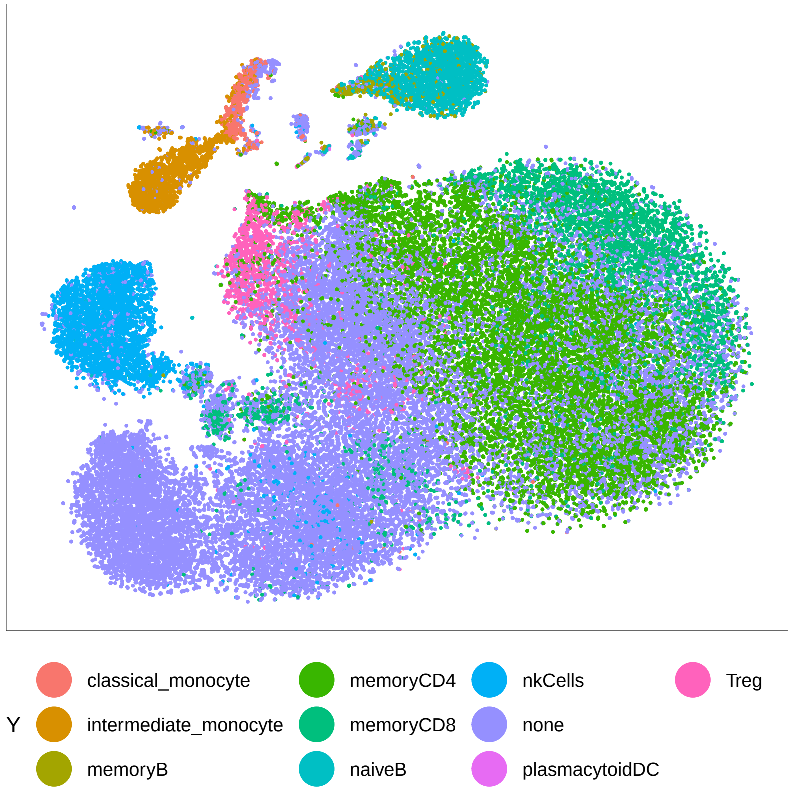

Supplementary Figure 4. After imputation by ALRA, positive and negative markers can be used to identify known cell types in peripheral blood mononuclear cells (PBMCs).

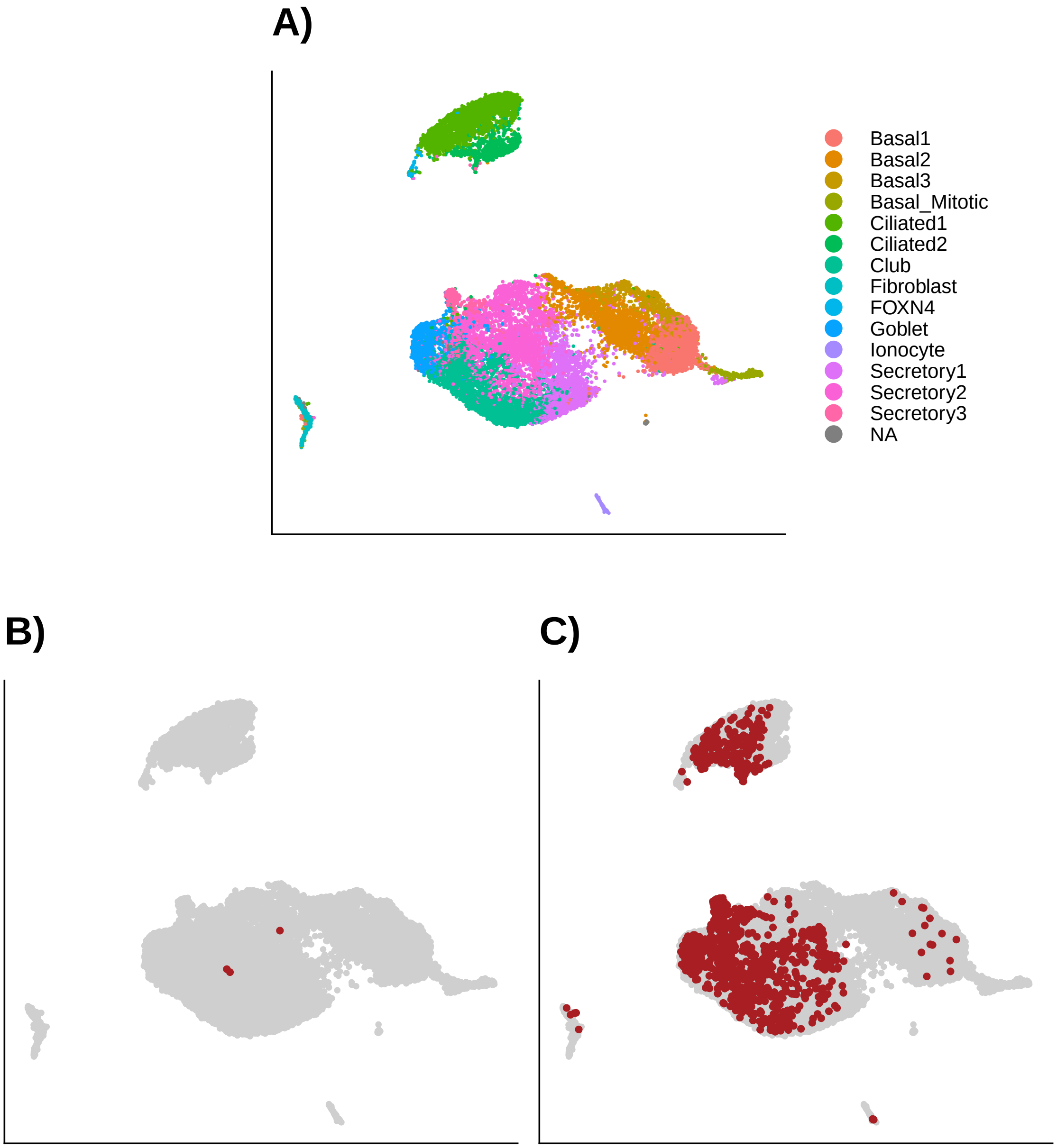

Supplementary Figure 5. Imputation of human bronchial cells. In the original dataset (A, colored by cell types), only 3 cells are ACE2+TMPRSS2+FURIN+ (B), whereas after imputation by ALRA 1,309 triple-positive cells are identified (C).

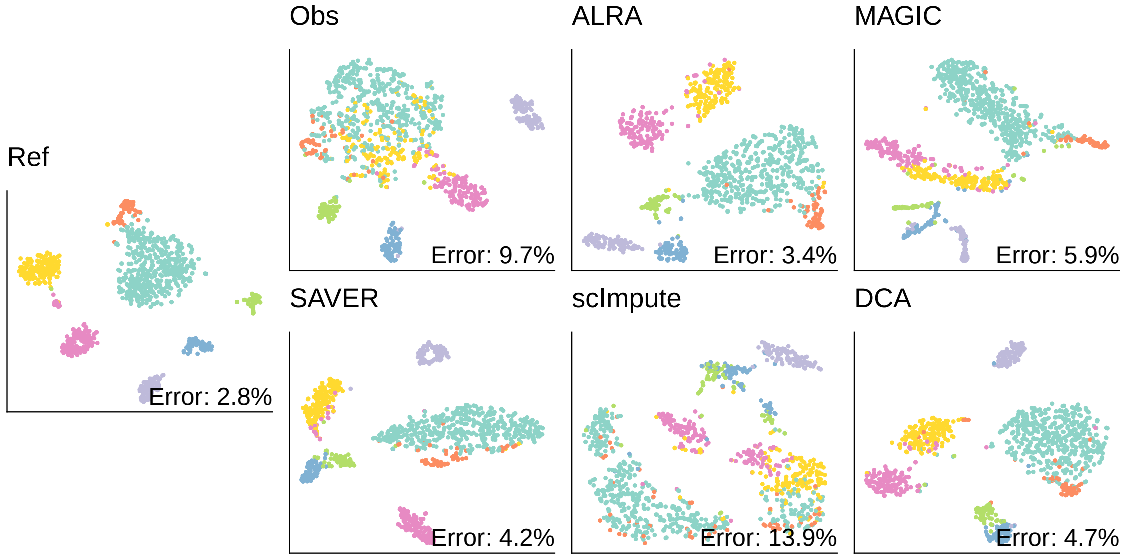

Supplementary Figure 6. Impact of imputation on t-SNE of downsampled dataset from Baron et al. The t-SNE of the original dataset (Ref) is compared to that of the downsampled data (Obs) and imputation by various methods.

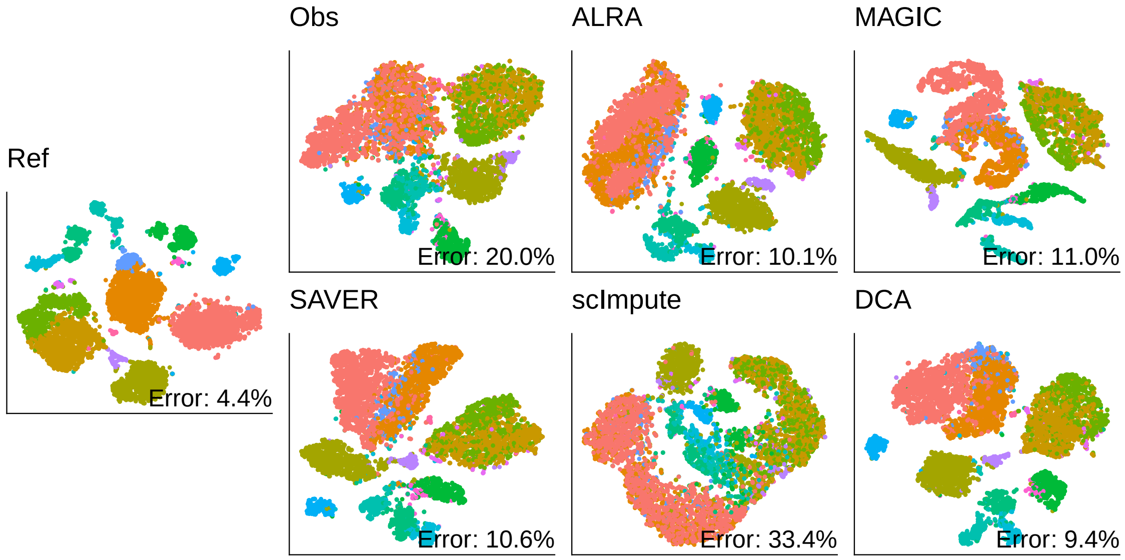

Supplementary Figure 7. Impact of imputation on t-SNE of downsampled dataset from Chen et al. The t-SNE of the original dataset (Ref) is compared to that of the downsampled data (Obs) and imputation by various methods.

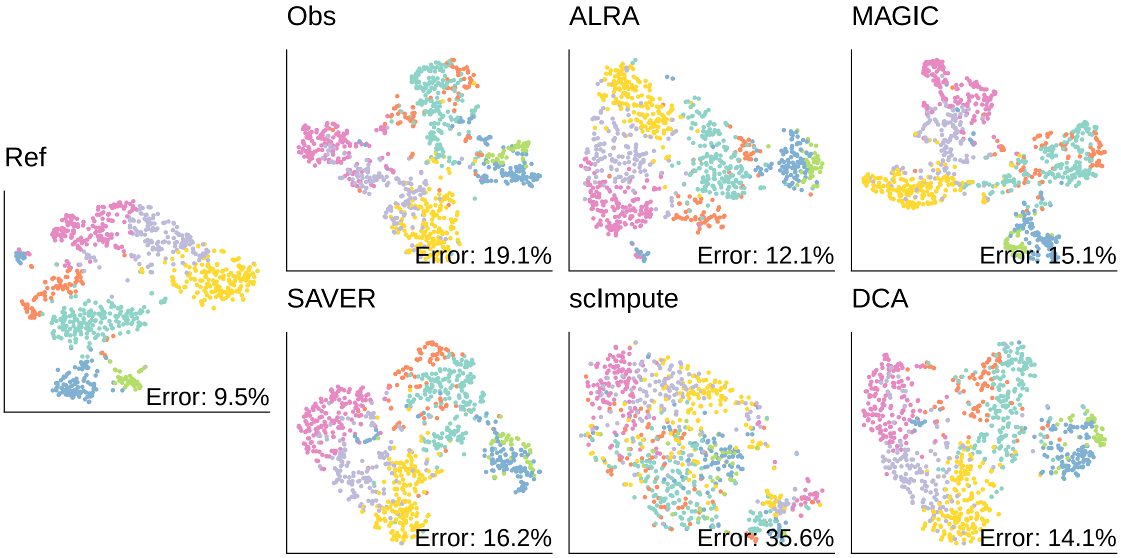

Supplementary Figure 8. Impact of imputation on t-SNE of downsampled dataset from La Manno et al. The t-SNE of the original dataset (Ref) is compared to that of the downsampled data (Obs) and imputation by various methods.

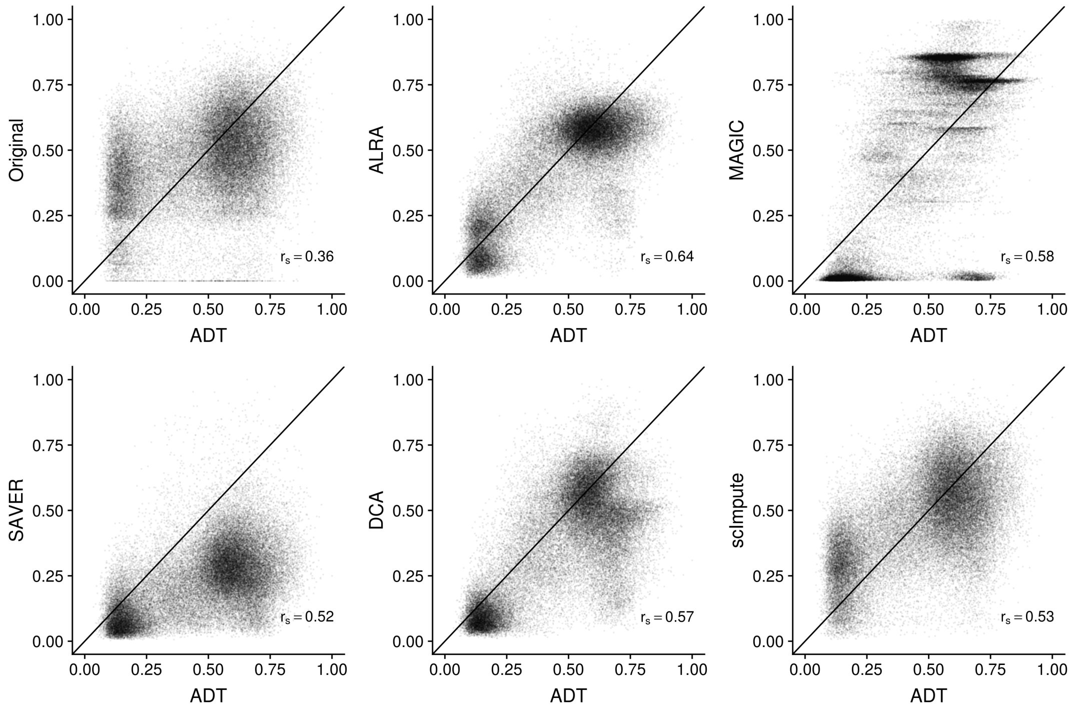

Supplementary Figure 9. Recovering true distances between points. Using CITE-seq data with measurements of both gene and protein expression of the same cells, we compute the pairwise distances between cells on the gene expression and on the protein expression data. We plot the normalized distances between cells in gene expression data before and after imputation against the distances between cells in the protein expression data. A random subset of 50,000 cell-cell distances is shown. Spearman's rho between the pairwise distances is also calculated.

A)

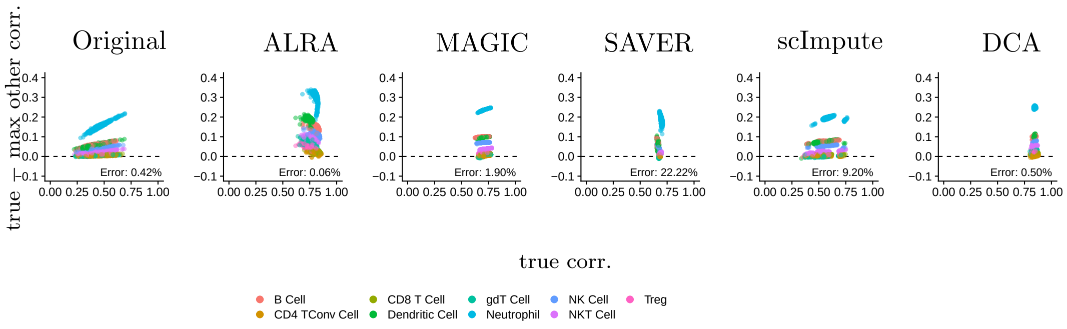

B)

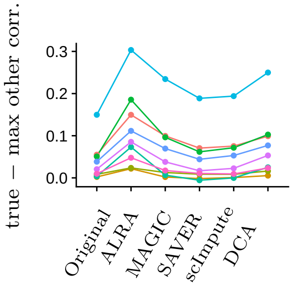

Supplementary Figure 10. Validating recovered expression profiles in simulation. A) Each cell is simulated from a bulk RNA-seq profile. We compute the correlation of each simulated cell with the bulk RNA-seq profiles, before and after imputation. The "true correlation" on the X-axis is the correlation of the cell with its corresponding bulk RNA-seq profile. The Y axis is the difference between this "true correlation" and the maximum correlation of the cell with bulk profiles it was not generated from. If cells were labeled by the bulk RNA-seq profile they are most correlated with, then cells below the 0-line on the Y-axis would be misclassified. B) Y axis in (A) averaged over cells for each population.

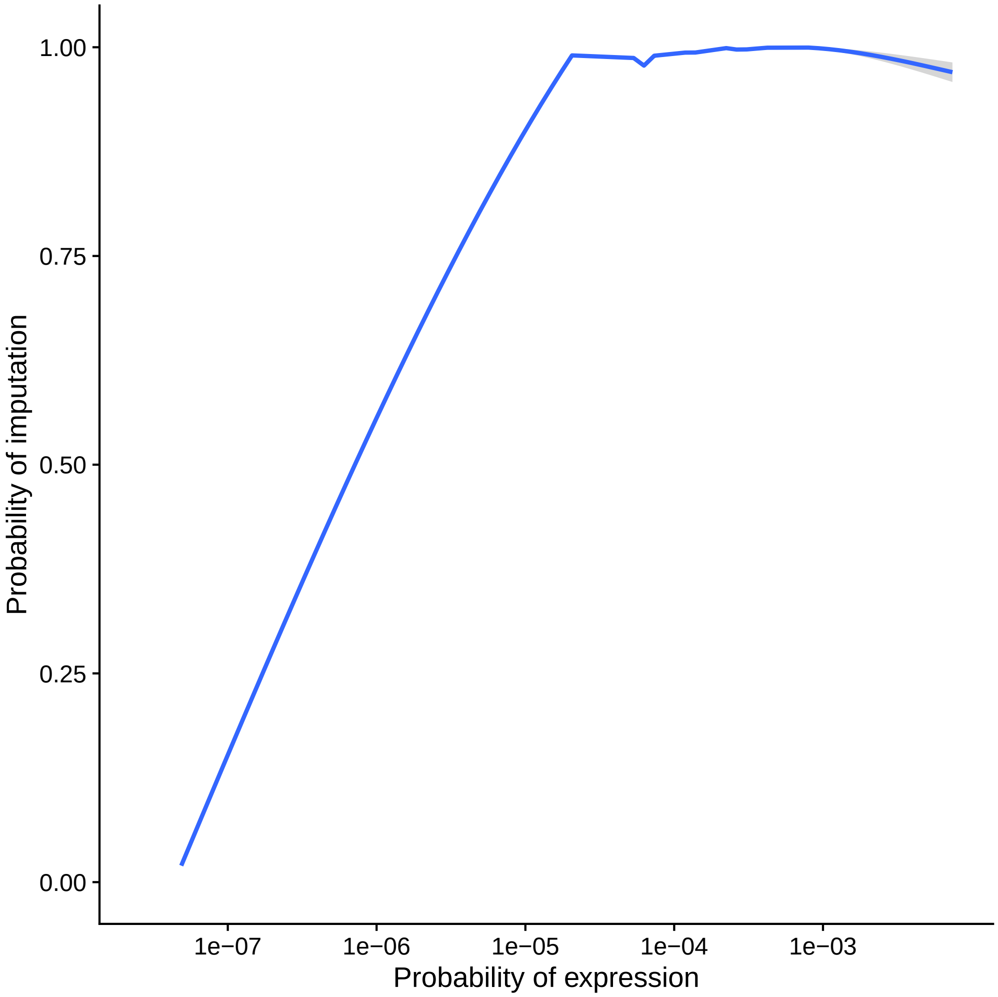

Supplementary Figure 11. Probability of imputing a technical zero as a function of its unobserved expression value. Each cell is sampled from a multinomial parameterized by a bulk RNA-seq profile normalized to be a probability vector. That is, the probability of expressing a gene in any given cell is given by its expression in the corresponding bulk profile divided by the total number of reads in that profile. The probability of recovering a non-zero value is shown as a function of this underlying probability of expression.

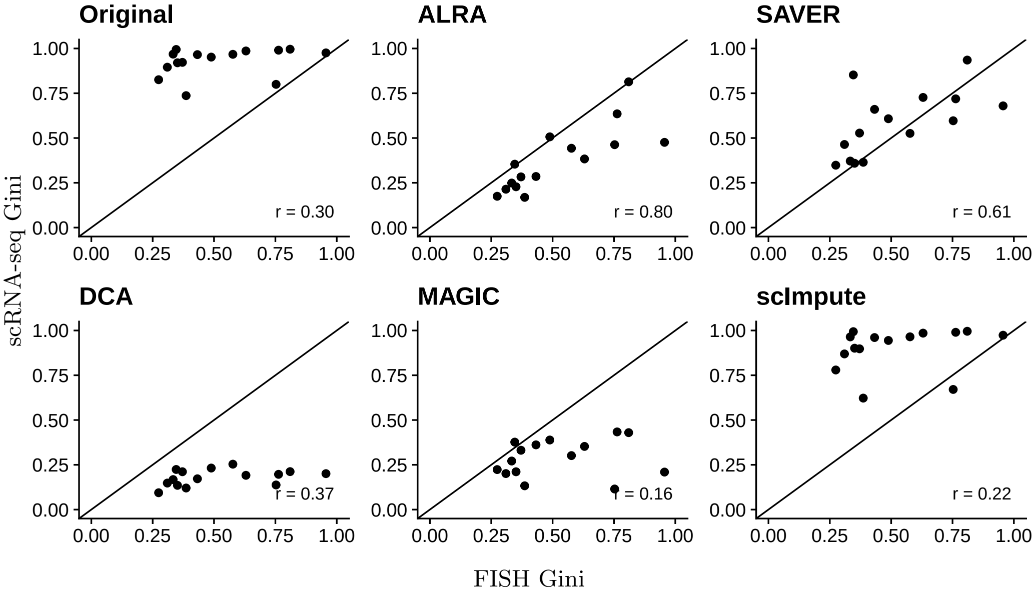

Supplementary Figure 12. Validating recovered expression profiles. Gini coefficient for 15 genes computed from scRNA-seq and FISH measurements of the same melanoma cell line.

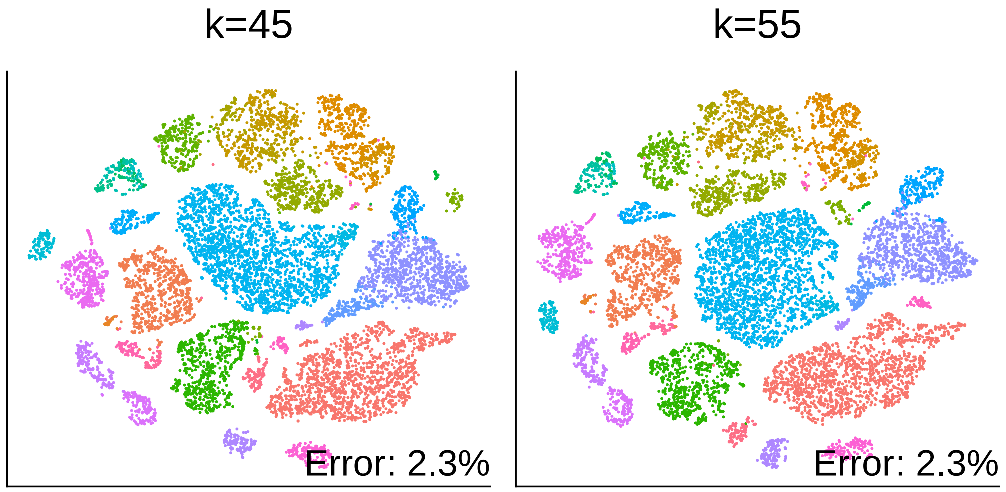

Supplementary Figure 13. The effect of imputation on separation of previously annotated cell types in mouse visual cortical cells from Hrvatin et al. is not sensitive to the choice of k.

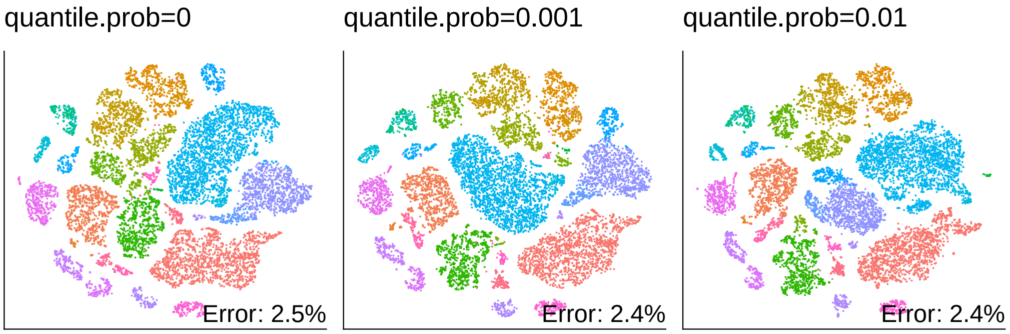

Supplementary Figure 14. The effect of imputation on separation of previously annotated cell types in mouse visual cortical cells from Hrvatin et al. is not sensitive to realistic choices of the quantile threshold p.

A

		Subspace Angle					
**Dataset**	**k**	**Observed**	**ALRA**	**MAGIC**	**SAVER**	**DCA**	**scImpute**
Baron et al.	6	0.34	0.25	0.28	0.24	0.25	0.37
Chen et al.	13	0.30	0.17	0.18	0.19	0.17	0.40
La Manno et al.	7	0.36	0.24	0.31	0.3	0.25	0.64
Zeisel et al.	13	0.21	0.15	0.19	0.19	0.18	0.47

B

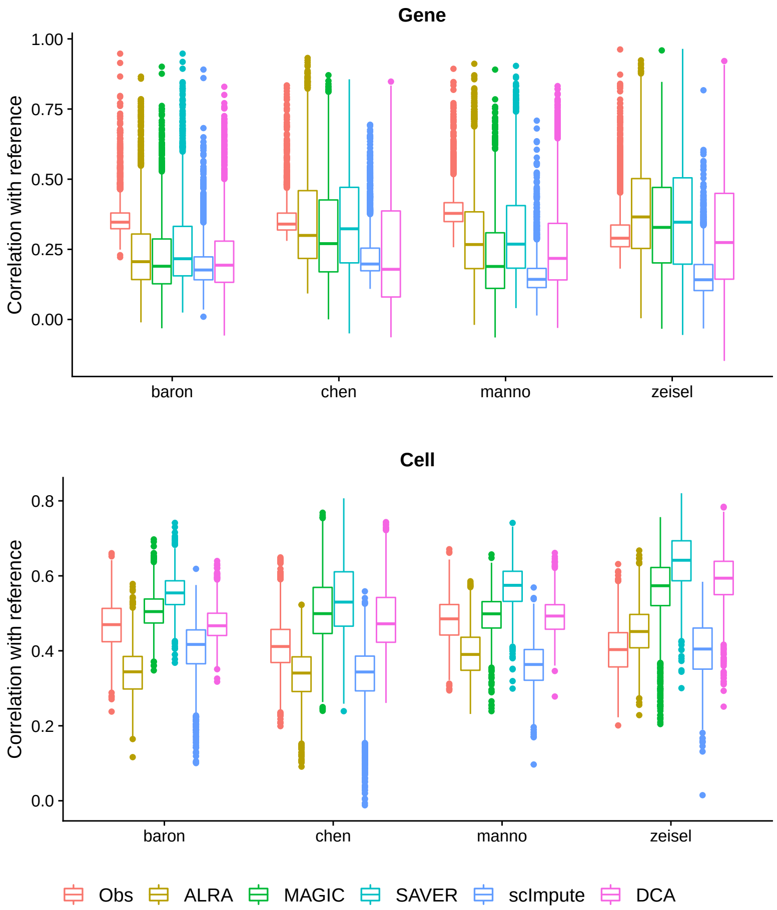

Supplementary Figure 15. Imputation after downsampling. (A) After downsampling each dataset, the subspace angle between the first k principal components of the original matrix with each of the imputed matrices. (B) Spearman's correlation between cells (genes) after imputation and cells (genes) in the original. For the gene correlation panel, n = 2284, 2159, 2059, 3529 genes were used to generate each boxplot in the Baron, Chen, Manno, Zeisel datasets. For the cell correlation panel, n = 1076, 7712, 947, 1799 cells were used to generate each boxplot in the Baron, Chen, Manno, Zeisel datasets. Data are presented in the boxplots as follows: center is data median; lower box bound is 25% quantile; upper box bound is 75% quantile; minima (lower whisker) is smallest observation greater than or equal to lower box bound - 1.5 * IQR; maxima (upper whisker) is largest observation less than or equal to upper box bound + 1.5 * IQR. IQR stands for interquartile range.

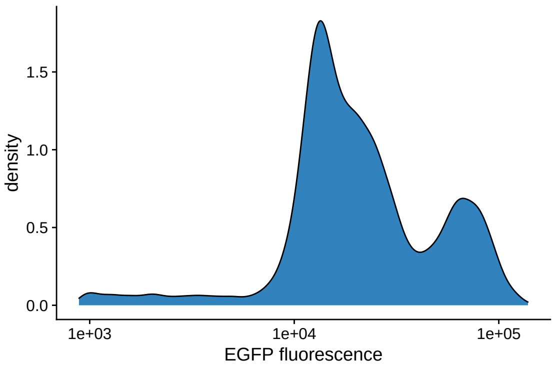

Supplementary Figure 16. Analysis of Pdgfra expression levels by flow cytometry in intestinal fibroblasts isolated from Pdgfra$^{EGFP/+}$ knockin mice, indicating that there are two populations of cells, based on level of Pdgfra expression. Only cells (n = 32667) with non-zero expression of Pdgfra are shown.

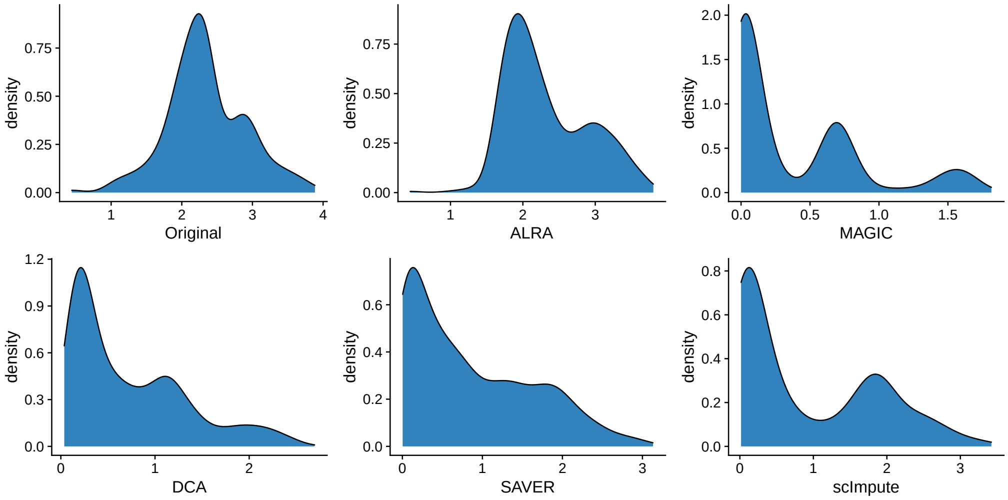

Supplementary Figure 17. Pdgfra expression in a scRNA-seq dataset of 1257 non-epithelial intestinal cells after completion by various methods. ALRA best preserves the true distribution of Pdgfra expression, showing two distinct populations.

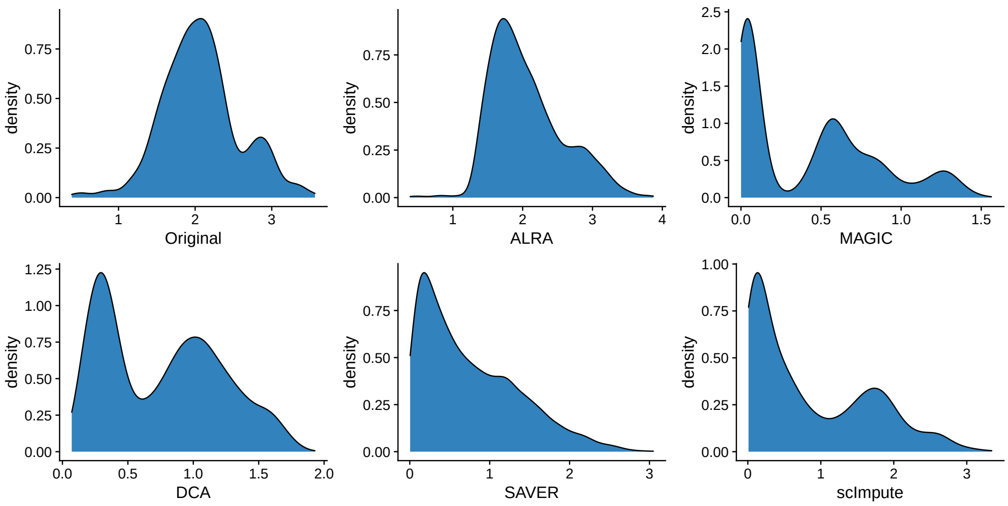

Supplementary Figure 18. Pdgfra expression in a biological replicate with 4113 cells. The two distinct populations are only obvious in ALRA, but it is not as clearly separated as in Supplemental Figure 16. Only cells with non-zero expression of Pdgfra are shown.

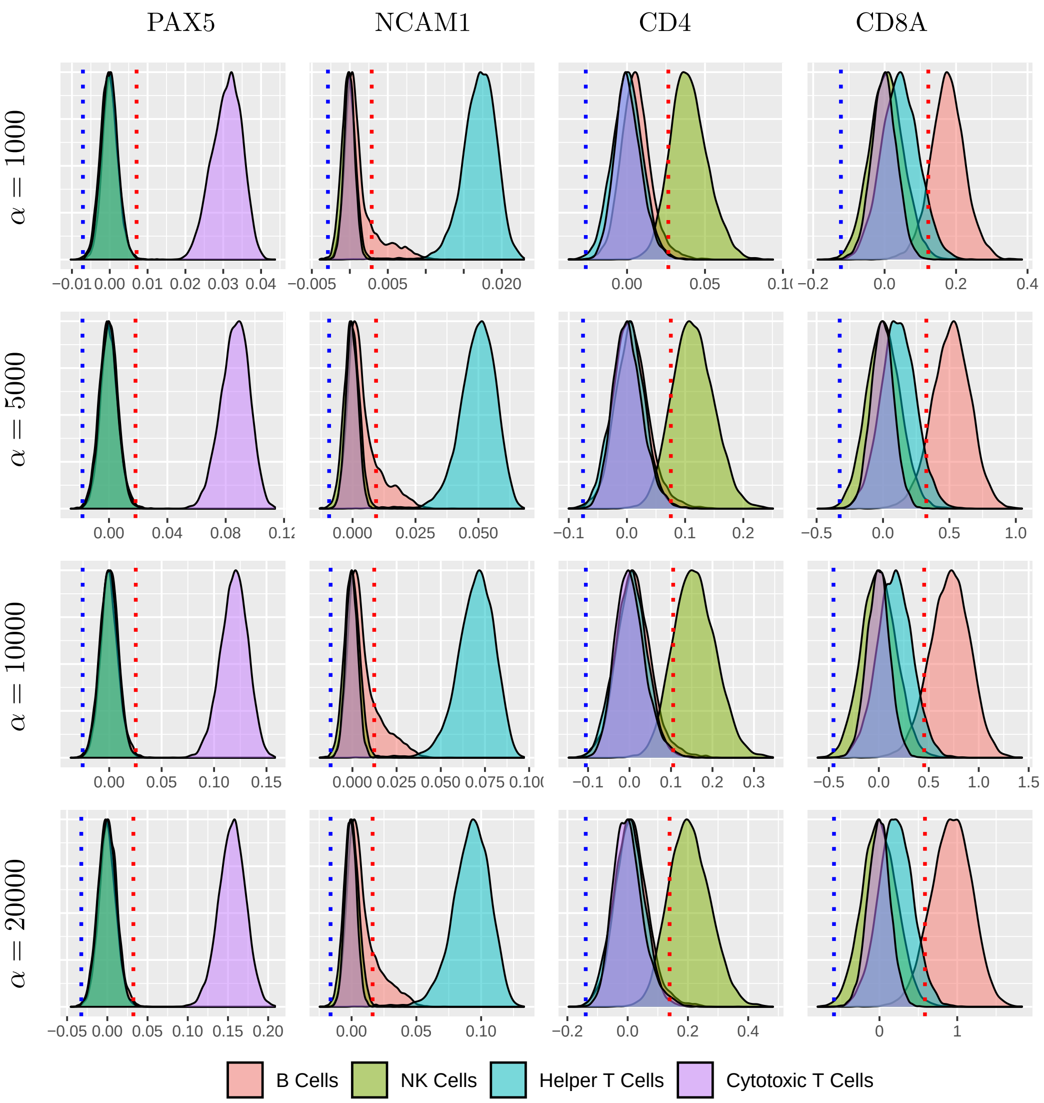

Supplementary Figure 19. Low-rank approximation of the purified PBMC dataset with different choice of scaling factors. The expression values were normalized as $\log(\frac{\alpha}{s_j} \cdot x_{ij} + 1)$, where α is the scaling factor and s_j is the library size for the jth cell. $\alpha = 10000$ corresponds to Figure 2A.

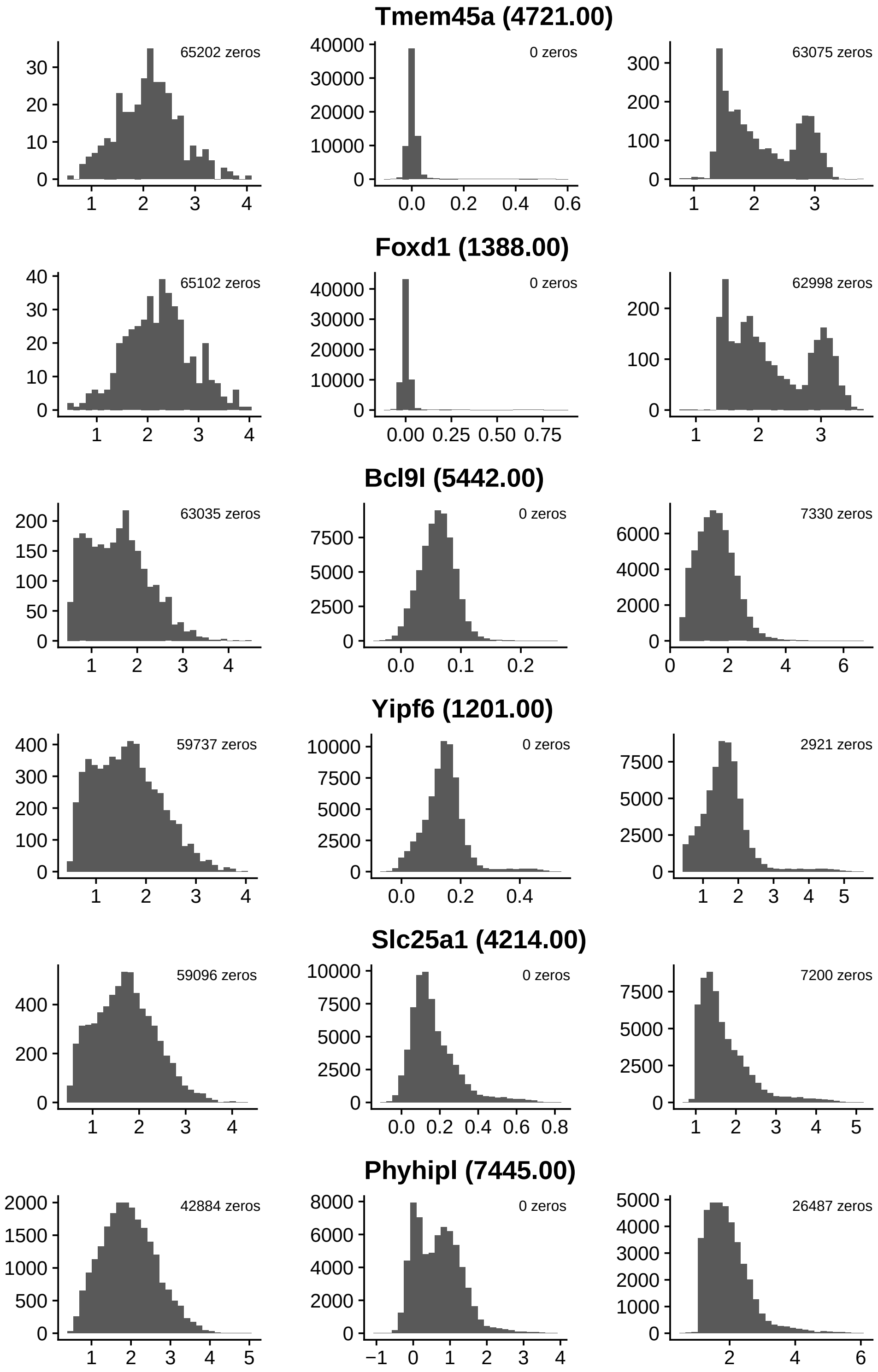

Supplementary Figure 20. Distributions of six randomly chosen variable genes with few excess zeros (EZs) from the mouse visual cortex cells of Hrvatin et al. before imputation (left), after low rank approximation (center), and after thresholding and scaling (right). Only non-zero values are plotted; the number of zeros is reported in the upper right corner.

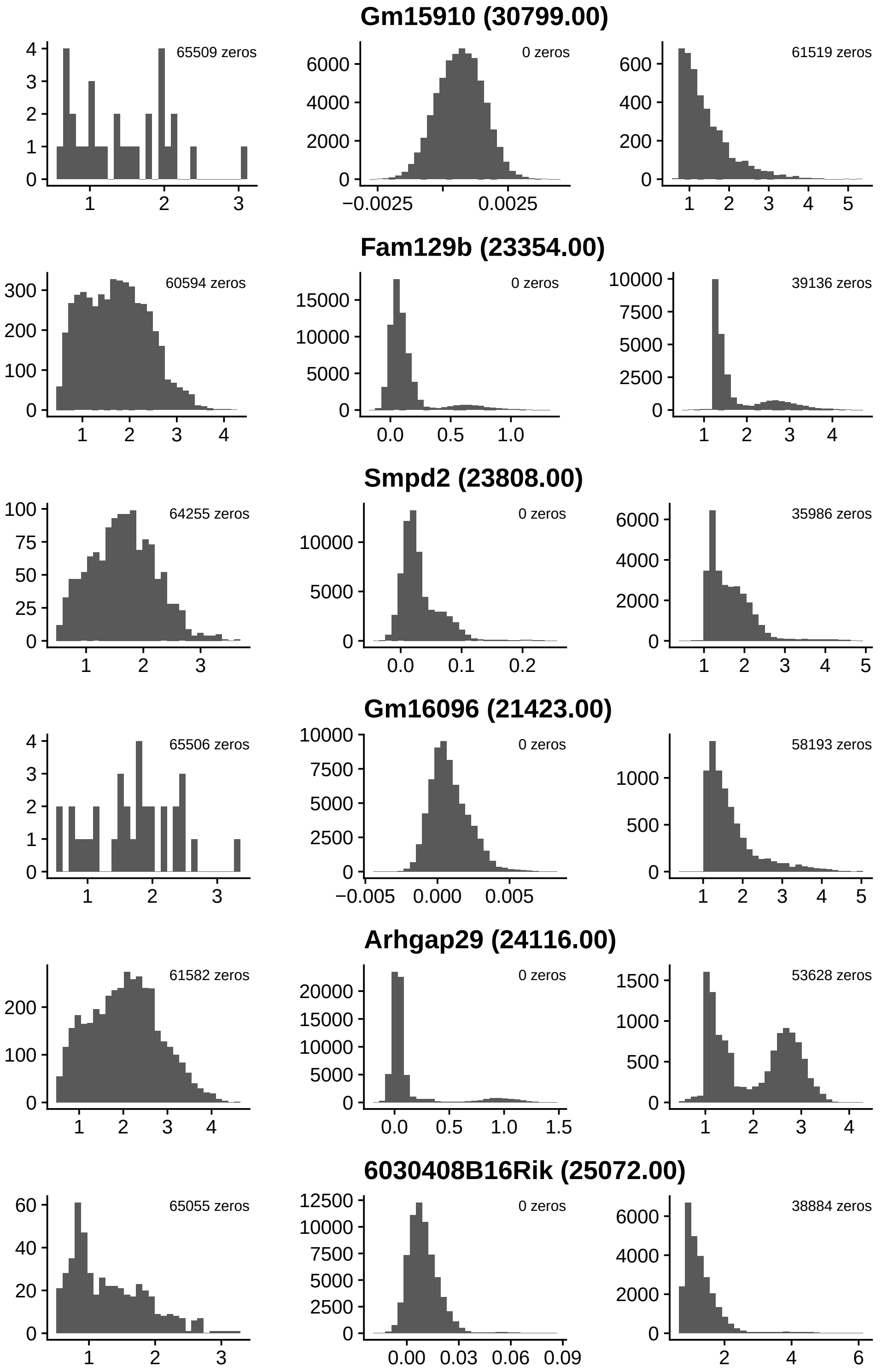

Supplementary Figure 21. Distributions of six randomly chosen variable genes with many excess zeros (EZs) from the mouse visual cortex cells of Hrvatin et al. before imputation (left), after low rank approximation (center), and after thresholding and scaling (right). Only non-zero values are plotted; the number of zeros is reported in the upper right corner.

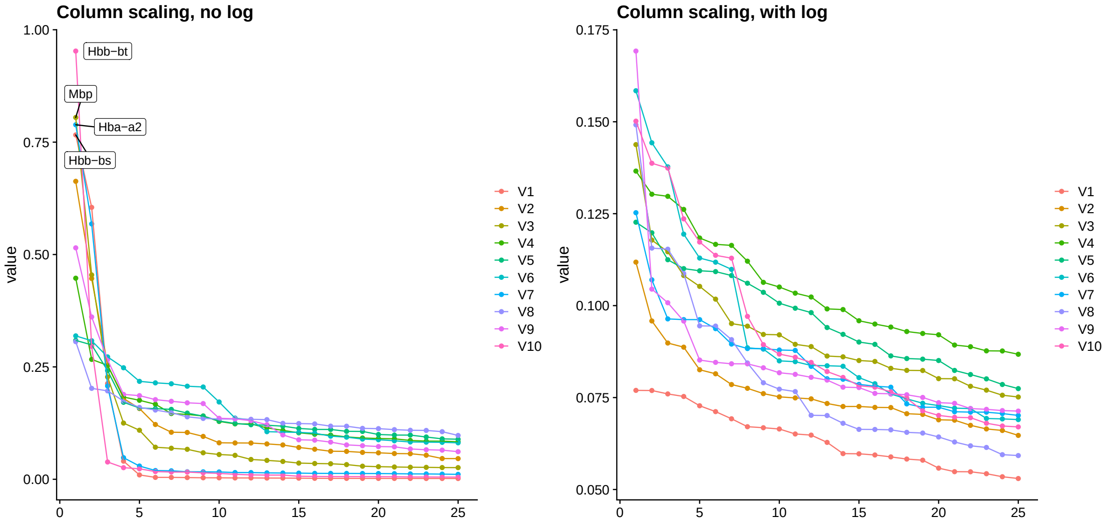

Supplementary Figure 22. Effect of log-normalization on right singular vectors in Hrvatin et al. The largest values of the top ten right singular vectors are shown in order from largest to small. Without log-normalization (left), each singular vector is dominated by only 1-3 genes. In contrast, with log-normalization, the mass of each singular vector is spread among a much larger number of genes.

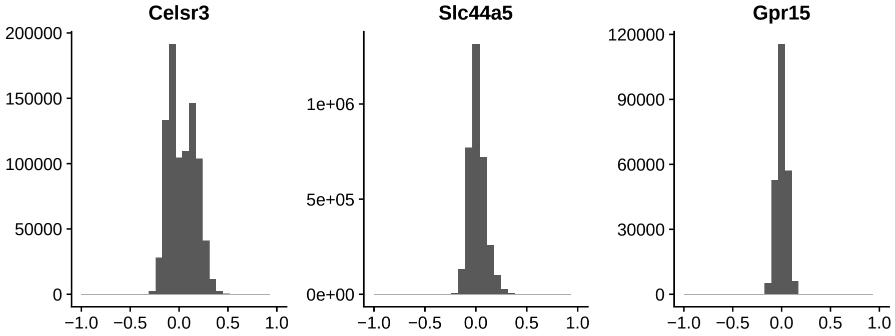

Supplementary Figure 23. Distribution of sample correlations ρ_i between entries corresponding to biological zeros in each of three randomly chosen gene.

4. Supplementary References

[1] Francis J Anscombe The transformation of Poisson, binomial and negative-binomial data, Biometrika, vol 35, no. 3/4, pp. 246–254. 1948

[2] Afonso S. Bandeira and Ramon van Handel, sharp non-asymptotic bounds on the norm of random matrices with independent entries, the Annals of Probability, vol 44, no. 4, pp. 2479–2506.

[3] Stephane Boucheron, Gabor Lugosi and Pascal Massart, *concentration inequalities, a non-asymptotic theory of independence,* oxford university press (2013).

[4] Iain M. Johnstone and Boaz Nadler, Roy's largest root test under rank-one alternatives, Biometrika **104** (2017), no. 1, 181–193.

[5] Ilse Ipsen and Boaz Nadler, Refined perturbation bounds for eigenvalues of Hermitian and non-Hermitian matrices, SIAM Journal on Matrix Analysis and Applications, **31** (2009) no. , 40–53.

[6] Tosio Kato. Perturbation theory for linear operators. springer science & business media (2013) 132.

[7] Erich L. Lehmann. Elements of large-sample theory. springer science & business media (2004).

[8] Boaz Nadler, Finite sample approximation results for principal component analysis: a matrix perturbation approach, the Annals of Statistics **36** (2008), no. 6, 2791–2817.

[9] F William Townes, Stephanie C. Hicks, Martin J. Aryee, and Rafael A. Irizarry, Feature selection and dimension reduction for single cell rna-seq based on a multinomial model, bioRxiv (2019), 574574.

[10] Roman Vershynin, Introduction to the non-asymptotic analysis of random matrices, Chapter 5 of *Compressed Sensing: Theory and Applications*, Yonina Eldar and Gitta Kutyniok, editors, Cambridge University Press, 2012. pp. 210–268.
