# Supplementary material for: Zero-preserving imputation of single-cell RNA-seq data
Source: Nat Commun. 2022 Jan 11;13:192. doi: 10.1038/s41467-021-27729-z (PMC8752663; doi:10.1038/s41467-021-27729-z)
Supplement: Supplementary file 3 — Reporting Summary [file 41467_2021_27729_MOESM3_ESM.pdf]

nature research

Corresponding author(s): Yuval Kluger

Last updated by author(s): Oct 26, 2021

Reporting Summary

Nature Research wishes to improve the reproducibility of the work that we publish. This form provides structure for consistency and transparency in reporting. For further information on Nature Research policies, see Authors & Referees and the Editorial Policy Checklist.

Statistics

For all statistical analyses, confirm that the following items are present in the figure legend, table legend, main text, or Methods section.

n/a	Confirmed	
☐	☒	The exact sample size (n) for each experimental group/condition, given as a discrete number and unit of measurement
☐	☒	A statement on whether measurements were taken from distinct samples or whether the same sample was measured repeatedly
☐	☒	The statistical test(s) used AND whether they are one- or two-sided<br>*Only common tests should be described solely by name; describe more complex techniques in the Methods section.*
☒	☐	A description of all covariates tested
☒	☐	A description of any assumptions or corrections, such as tests of normality and adjustment for multiple comparisons
☒	☐	A full description of the statistical parameters including central tendency (e.g. means) or other basic estimates (e.g. regression coefficient) AND variation (e.g. standard deviation) or associated estimates of uncertainty (e.g. confidence intervals)
☐	☒	For null hypothesis testing, the test statistic (e.g. F, t, r) with confidence intervals, effect sizes, degrees of freedom and P value noted<br>*Give P values as exact values whenever suitable.*
☒	☐	For Bayesian analysis, information on the choice of priors and Markov chain Monte Carlo settings
☒	☐	For hierarchical and complex designs, identification of the appropriate level for tests and full reporting of outcomes
☒	☐	Estimates of effect sizes (e.g. Cohen's d, Pearson's r), indicating how they were calculated

Our web collection on statistics for biologists contains articles on many of the points above.

Software and code

Policy information about availability of computer code

Data collection	Single-cell RNA-seq of mouse intestinal fibroblasts was the only data collected for this study, and it will be deposited to Gene Expression Omnibus upon acceptance. All public datasets used (with links and accession codes) are reported in the manuscript. No software was used for data collection.
Data analysis	FIt-SNE v1.1.0, rsvd 1.0.0, rpca-mkl (commit:d44e3b0), R v3.4.4, Rmagic v2.0.3, SAVER v1.1.1, scImpute v0.0.9, DCA 0.2.2, custom codes to analyze data is deposited at https://github.com/KlugerLab/ALRA-paper

For manuscripts utilizing custom algorithms or software that are central to the research but not yet described in published literature, software must be made available to editors/reviewers. We strongly encourage code deposition in a community repository (e.g. GitHub). See the Nature Research guidelines for submitting code & software for further information.

Data

Policy information about availability of data

All manuscripts must include a data availability statement. This statement should provide the following information, where applicable:

- Accession codes, unique identifiers, or web links for publicly available datasets
- A list of figures that have associated raw data
- A description of any restrictions on data availability

The purified PBMCs of Zheng et al. can be downloaded from 10x genomics website (https://www.10xgenomics.com/resources/datasets/). Bulk RNA-seq data from ImmGen (Heng et al.) can be downloaded from NCBI BioProject PRJNA281360. Other datasets can be downloaded from NCBI Gene Expression Omnibus: Hoek et al. (GSE64655), Stoeckius et al. (GSE100866), Gupta et al. (GSE122043), Hrvatin et al. (GSE102827), Tasic et al. (GSE115746), Torre et al. (GSE99330). The datasets from Chen et al. (GSE87544), Baron et al. (GSM2230757), La Manno et al. (GSE76381), Zeisel et al. (https://linnarssonlab.org/cortex) were used as preprocessed by Huang et al. and can be downloaded from https://github.com/mohuangx/SAVER-paper. The mouse intestinal fibroblast data is available at NCBI GEO, accession number GSE185638.

Field-specific reporting

Please select the one below that is the best fit for your research. If you are not sure, read the appropriate sections before making your selection.

☒ Life sciences ☐ Behavioural & social sciences ☐ Ecological, evolutionary & environmental sciences

For a reference copy of the document with all sections, see nature.com/documents/nr-reporting-summary-flat.pdf

Life sciences study design

All studies must disclose on these points even when the disclosure is negative.

Sample size

ALRA was applied to eleven existing datasets. In two datasets, we showed that ALRA preserves biological zeros. In six datasets, we showed that ALRA improves separation between known cell types. In three datasets, we showed that ALRA's imputed values are consistent with outside knowledge.
In the mouse intestinal fibroblast data, one preparation of Drop-seq collection was sequenced. No statistical methods were used to determine sample size. The sample size (n = 2-3 mice) was sufficient because of the minimal variation observed and consistent with the known literature.

Data exclusions

Low quality cells were excluded as follows, using criteria that were established prior to the analysis:
Zheng et al.: cells with expression in less than 400 genes were filtered out, genes with expression in less than 100 cells were filtered out.
Tasic et al.: cells labeled as 'Low Quality', 'Doublet', 'Batch Grouping', or 'High Intronic' in the metadata provided by the authors were filtered.
Stoeckius et al. (CITE-seq PBMC dataset): cells for which more than 90% of UMIs came from mouse genes were removed. Filtered genes with expression in less than 10 cells.
Stoeckius et al. (CITE-seq CBMC dataset): cells for which more than 90% of UMIs came from mouse genes were removed.
Gupta et al.: filter cells with low depth (< 1000 genes with non-zero expression), select only dermal cells (after clustering, keep clusters with ratio of cells expression Col1a1 > 0.85).
Mouse intestinal fibroblast data: cells with lower than 1000 nUMIs were removed.

The datasets from Baron et al., Chen et al., La Manno et al., Zeisel et al., Hrvatin et al., and Torre et al. were used as preprocessed by Huang et al. The filtering criteria below is quoted from their reporting summary:
Baron: genes with mean expression less than 0.001 and non-zero expression in less than 3 cells were filtered out.
Chen: cells with library size greater than 15,000 were filtered out. Genes with mean expression less than 0.0002 and non-zero expression in less than 5 cells were filtered out.
La Manno: genes with mean expression less than 0.001 and non-zero expression in less than 3 cells were filtered out.
Hrvatin et al.: genes with expression less than 0.0003 or non-zero expression in less than four cells were filtered out.
Torre et al.: genes with mean expression less than 0.1 as well as cells with library size less than 500 or greater than 20,000 were removed.

Replication

The code for all analyses is published and running that code produces the results. All experimental findings in mice were validated in independent experiments.

Randomization

Mice were randomly selected for experimental analyses.

Blinding

There was no blinding. The analyses performed do not involve the evaluation of any subjective parameters.

Reporting for specific materials, systems and methods

We require information from authors about some types of materials, experimental systems and methods used in many studies. Here, indicate whether each material, system or method listed is relevant to your study. If you are not sure if a list item applies to your research, read the appropriate section before selecting a response.

Materials & experimental systems

n/a	Involved in the study	
☒	☐	Antibodies
☒	☐	Eukaryotic cell lines
☒	☐	Palaeontology
☐	☒	Animals and other organisms
☒	☐	Human research participants
☒	☐	Clinical data

Methods

n/a	Involved in the study	
☒	☐	ChIP-seq
☐	☒	Flow cytometry
☒	☐	MRI-based neuroimaging

Animals and other organisms

Policy information about studies involving animals; ARRIVE guidelines recommended for reporting animal research

Laboratory animals

Species: Mus musculus, strain: C57BL/6J, age: 2 months-old. The mice were bred in the Yale Animal Resources Center Facilities

Laboratory animals

under specific pathogen-free conditions and maintained on a C57BL/6J genetic background. Mice were housed in standard cages, on a 12-hour day/night cycle and were fed a standard rodent chow. All animal experimentation was performed in compliance with Yale Institutional Animal Care and Use Committee protocols.

Wild animals

Provide details on animals observed in or captured in the field; report species, sex and age where possible. Describe how animals were caught and transported and what happened to captive animals after the study (if killed, explain why and describe method; if released, say where and when) OR state that the study did not involve wild animals.

Field-collected samples

For laboratory work with field-collected samples, describe all relevant parameters such as housing, maintenance, temperature, photoperiod and end-of-experiment protocol OR state that the study did not involve samples collected from the field.

Ethics oversight

All animal experimentation at Yale was performed in compliance with Yale Institutional Animal Care and Use Committee protocols.

Note that full information on the approval of the study protocol must also be provided in the manuscript.

Flow Cytometry

Plots

Confirm that:

☒ The axis labels state the marker and fluorochrome used (e.g. CD4-FITC).

☒ The axis scales are clearly visible. Include numbers along axes only for bottom left plot of group (a 'group' is an analysis of identical markers).

☐ All plots are contour plots with outliers or pseudocolor plots.

☒ A numerical value for number of cells or percentage (with statistics) is provided.

Methodology

Sample preparation

The intestine was dissected, flushed, opened longitudinally and then cut into 1 cm pieces. The tissues were incubated in HBSS containing 1 mM EDTA, 1 mM DTT, 0.2 % FBS, 4-5 times, 10 min each, at 37 °C, 200 rpm. Epithelial cells were released by vigorous shaking. After epithelial cell removal, the remaining stromal part of the intestine was incubated in DMEM 10% FBS containing Collagenase XI (300 units/ml, Sigma, C7657), Dispase II (0.1 mg/ml, Sigma, D4693) and DNase II Type V (50 units/ml, Sigma, D8764) for 1 h, at 37 oC, 200 rpm. Cells released after vigorous shaking were passed through a 70 m strainer and washed with 2% sorbitol. Such cell preparations were directly processed by flow cytometry.

Instrument

BD LSR-II equipped with FACSDiva 7 software

Software

Data were acquired with the BD FACSDiva 7 software

Cell population abundance

The population of interest includes all cells acquired

Gating strategy

FSC-A, FSC-H singlets >> FSC-A, SSC-A live cells >> Pdgfra-EGFP+

☐ Tick this box to confirm that a figure exemplifying the gating strategy is provided in the Supplementary Information.
